# Complementary MR measures of white matter and their relation to cardiovascular health and cognition

**DOI:** 10.1038/s41598-025-13610-2

**Published:** 2025-08-07

**Authors:** Petar P. Raykov, Marta Correia, Kamen Tsvetanov, Rafael N. Henriques, Alberto Del Cerro-León, Matthew Bracher-Smith, Valentina Escott-Price, Yordan P. Raykov, Richard N. Henson

**Affiliations:** 1 Medical Research Council Cognition and Brain Sciences Unit, University of Cambridge, 15 Chaucer Road, Cambridge, CB2 7EF UK; 2 Department of Psychiatry, University of Cambridge, Cambridge, UK; 3 Champalimaud Research, Champalimaud Foundation, Lisbon, Portugal; 4 Center for Cognitive and Computational Neuroscience (C3N), Department of Experimental Psychology, Universidad Complutense de Madrid (UCM), Madrid, Spain; 5 School of Medicine, Division of Psychological Medicine and Clinical Neurosciences, University of Cardiff, Cardiff, UK; 6 School of Mathematical Sciences, University of Nottingham, Nottingham, UK

**Keywords:** Psychology, Human behaviour, Cognitive ageing, Cognitive neuroscience

## Abstract

The microstructural and macrostructural integrity of white matter (WM) underpins efficient brain function, and is known to decline with age and vascular burden. Key aspects of WM health include axonal fibre density, myelination, free-water content, and the presence of tissue damage or lesions. Magnetic Resonance Imaging (MRI) offers multiple complementary sequences to non-invasively estimate these properties in vivo. For example, diffusion-weighted imaging (DWI) provides sensitive measures of microstructure, while T1-weighted and T2-weighted MRI can estimate total WM volume and hyper-intensities, and magnetisation transfer imaging (MT) and T1:T2 ratios can indicate myelin content. In this study, we leveraged all of these MRI-derived measures in a large population-based cohort (Cam-CAN) to identify latent WM factors and test how these factors relate to cardiovascular health and cognitive performance. Among 11 commonly-used WM metrics [Fractional Anisotropy (FA); Mean Signal Diffusion (MSD); Mean Signal Kurtosis (MSK); Neurite Density Index (NDI); fibre Orientation Dispersion Index (ODI); Free water volume faction (F_iso_); spread of Mean Signal Diffusivity values (MSDvar); Magnetisation Transfer Ratio (MTR); T1:T2 ratio; volume of White Matter Hyper-Intensities (WMHI); White Matter Volume (WMV)], latent factor analysis showed that four factors were needed to explain 89% of the variance, which we interpreted in terms of (1) fibre density/myelination, (2) free-water / tissue damage, (3) fibre-crossing complexity and (4) microstructural complexity. These factors showed distinct effects of age and sex. To test the validity of these factors, we related them to measures of cardiovascular health and cognitive performance. Specifically, we ran path analyses linking (1) cardiovascular factors to the WM factors, and (2) the WM factors to cognitive measures. Even after adjusting for age and sex, we found that a vascular factor related to pulse pressure predicted the WM factor capturing free-water/tissue damage, and that several WM factors made unique predictions for fluid intelligence and processing speed. Our results show that there is both complementary and redundant information across common MR measures of WM, and their underlying latent factors may be useful for pinpointing the differential causes and contributions of white matter health in aging.

**Supplementary Information:**

The online version contains supplementary material available at 10.1038/s41598-025-13610-2.

## Introduction

The structural integrity of white matter (WM)—including features such as fibre density, myelination and degree of tissue damage—is fundamental for rapid neural signalling, which is presumed to enable efficient cognitive performance. These aspects of WM health are known to decline across the adult lifespan, and are linked to slower processing speed, reduced fluid intelligence and neurodegenerative disease^1–11^. To characterise these properties in vivo, various Magnetic Resonance Imaging (MRI) contrasts can be used, each providing possibly complementary information. For instance, T1-weighted (T1w) and T2-weighted (T2w) images can be used to estimate macrostructural features such as total WM volume and the presence of WM Hyper-Intensities (WMHI); Magnetisation Transfer Ratio (MTR) and T1:T2 ratios have been argued to provide proxies for myelin content; and Diffusion-Weighted Imaging (DWI) can be used to estimate microstructural features like fibre density and dispersion. However, MRI studies often rely on a single metric, making it challenging to disentangle the unique and shared contributions of these properties to brain health and cognition^12^. Here, we take advantage of a broad set of MRI-derived WM measures in a large life-span cohort (650 adults aged 18–89 from the Cam-CAN cohort) to address two questions: (1) how these measures cluster into latent factors reflecting distinct aspects of WM health, and (2) how these factors relate to cardiovascular risk and cognitive abilities.

Ageing is well-known to reduce the total volume of WM during the last decades of life^13^ in addition being associated with reductions in gray matter and changes in whole brain functional organisation^12,14,15^. These white matter volume (WMV) reductions could reflect the degree of axonal degeneration or demyelination^16^ and can be estimated by segmenting T1w images using automated techniques. Early MRI studies also observed bright regions on T2w images, which tend to become larger and more numerous with age. The prevalence of these “white matter hyper-intensities” (WMHI) has been associated with impaired cognition and increased risk of neurodegenerative disease^17–23^. They are thought to reflect macroscopic damage to the WM, often due to vascular pathology.

Other MR sequences have been developed to provide more direct measures of myelin content. For instance, magnetisation-transfer-weighting (MTw) relies on the magnetisation exchange between macromolecule-bound protons (in myelin, for example) and free water protons. The amount of magnetisation transferred between the two pools of protons can be quantified by computing the ratio between two images: one with and one without a RF saturation pulse. The resulting magnetisation-transfer ratio (MTR) is sensitive to the proportion of bound proteins, and hence thought to reflect myelin content. Indeed, the MTR is commonly used in the study of demyelination disorders such as multiple sclerosis^24,25^. However, the MTR is unlikely to be a selective measure of myelin, because it is also affected by iron concentration, inflammation as well as water content^26–30^.

Another approach to estimate myelin uses the ratio of signals from T1w and T2w images^31–33^. This relies on the premise that T1w and T2w contrasts are both sensitive to myelin content, but in the opposite manner, such that their ratio enhances myelin sensitivity (provided appropriate B0 corrections are applied to each image;^31,32)^. This measure has mainly been applied to study cortical myelin content, but some studies have also used it to examine integrity of WM tracts^32,34–43^. However, it is still unclear how the T1/T2 ratio in WM tracts relates to other WM measures. Indeed, some recent work suggests that the T1/T2 ratio in WM is not necessarily sensitive to myelin^44–46^.

Most recently, DWI has become the main technique to study WM and microstructural properties of the brain^45,46^. It measures the diffusion of water molecules in vivo, which happens at the micrometric scale during the duration of a typical scan, thereby providing information about microstructural alterations below the scale of the image voxels^49,50^. Indeed, previous work has argued that DWI provides the most sensitive index of WM alterations compared to more conventional structural MRI contrasts such as those described above^51–53^.

DWI provides multi-dimensional information, to which different mathematical models can be fitted in order to extract properties of the diffusion tensor, such as fractional anisotropy (FA) and mean signal diffusion (MSD), and including higher order moments, such as mean signal kurtosis (MSK)^54,55^. These have been indirectly related to the underlying WM microstructure^56–58^. Biophysical microstructural models have also been fitted in attempts to estimate parameters with a direct biological interpretation^59–61^. One of the most popular of these is the “Neurite Orientation Dispersion and Density Imaging” (NODDI) model^62^ which measures the neurite density index (NDI), fibre orientation dispersion index (ODI) and the free water volume fraction (F_iso_). More recently still, Baykara et al.^63^ suggested that the spread of MD values across voxels within WM tracts (a WM skeleton)—which they called the “peak width of skeletonised mean diffusivity” (PSMD) and shown to be particularly sensitive to age-related WM differences. This measure, like WMHI volume, captures WM damage that can, in principle, occur anywhere in the brain within the white matter skeleton.

While many studies have examined the effects of age on at least one of these MRI metrics of WM^5,6,9,11,57,62–69^ their simultaneous presence in a large cohort is rare. Furthermore, multiple WM metrics are often tested separately, rather than simultaneously, and their relative sensitivity to ageing and cognition has not been systematically explored. There are prior suggestions that they may be somewhat complementary. For example, Westlye et al. reported that the macrostructural property of total WM volume (WMV) was not highly correlated with the microstructural property of FA measured from DWI^71^. There are multiple potential reasons for this, such as FA and WMV being differentially sensitive to the presence of non-neuronal cells in WM, and/or the proportion of free water and/or the organisation of axons. Indeed, even within the domain of DWI, Henriques et al. used factor analysis to conclude that three factors under-pinned their six DWI metrics (FA, MSD, MSK from a tensor model, and NDI, ODI and F_iso_ from the NODDI microstructural model), indicating both shared but also unique variance explained by their combination^72^.

**Fig. 1:**
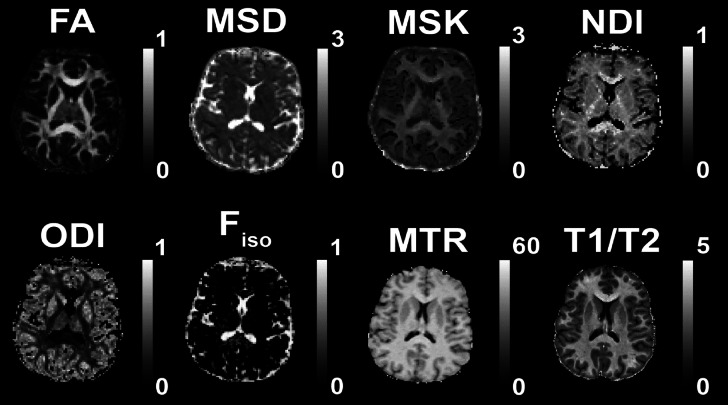
Example Brain maps of the 8 WM measures from a single subject aged 22 included in the analyses. We show a young participant’s data from 6 diffusion metrics (FA, MSD, MSK, NDI, ODI, F_iso_), plus the additional measures of MTR and T1/T2 ratio. Not shown are the raw T1w + T2w images from which total WM volume (TWM) was calculated, and the other two global measures of White Matter Hyperintensities (WMHI), which were computed across WM ROIs using the T1w and T2w images and the SAMSEG algorithm, and PSMD, which was computed across the WM ROIs using the DWI FA and MSD measures.

Here, we started with a similar dimension reduction approach, but now using a total of 11 whole-brain, WM measures: the 6 DWI metrics used by Henriques et al. plus a PSMD-like measure, together with WMV (T1w), WMHI (T2w), MTR and T1/T2 ratio^72^ (see Fig. 1). Apart from examining the relationship of all these measures and whether they can be sufficiently well represented by a small number of factors, we also examined whether the derived factors had convergent construct validity, in terms of being related to other biological and cognitive measures, namely cardiovascular health and cognitive performance.

The reason for focusing on cardiovascular health was because it has been suggested that WM is particularly vulnerable to hypoxia from reduced cerebral blood flow^73^. This is because the arterioles that supply oxygen to astrocytes and oligodendrocytes are narrow and long, making perfusion more difficult to maintain compared to perfusion in gray matter^74–77^. Indeed, recent work has shown that WM microstructural changes are associated with increased cardiovascular risk in middle and older adults^78–81^. Multiple cardiovascular measures have been used in previous studies, including heart-rate and heart-rate variability, body-mass index, static blood pressure, and pulse pressure – the difference between systolic and diastolic blood pressure. Most of these studies have linked a single cardiovascular measure to a single measure of WM^18,19,82–89^. Here, we utilised previous work in the Cam-CAN cohort by King and colleagues^90,91^ who showed that common cardiovascular measures can be captured by 3 distinct latent factors. More specifically, we examined how these 3 cardiovascular factors predicted our WM factors.

In terms of the consequences rather than causes of WM health, we examined how our WM factors related to several “fluid” cognitive measures, namely fluid intelligence, processing speed and episodic memory. These cognitive measures were first adjusted for age and sex. To additionally account for other individual differences in cognition in participants, we ran an additional control analysis where we tested the relationship between WM factors and cognition after adjusting for each individual’s polygenic propensity score for high general cognitive ability.

## Methods

### Ethics & inclusion statement

Approval for the Cam-CAN study was granted by the Research Ethics Committee of Cambridgeshire 2 (now known as East of England—Cambridge Central—reference number: 10/H0308/50). Prior to their involvement, participants provided written, informed consent. All experiments were performed in accordance with relevant guidelines and regulations.

### Participants and materials

We used neuroimaging data from Phase 2 (Arm 1) of the Cambridge Centre for Aging and Neuroscience (Cam-CAN, www.cam-can.org^92^, which included healthy individuals aged between 18 and 89, approximately half male/female. None of the participants had a current diagnosis of dementia or mild cognitive impairment, and scored 25 or higher on the mini mental state examination^93^. Participants were native English speakers, had no neurological disorders, and had normal or corrected to normal vision and hearing (for further details^92^. See Supplementary Table 1 for descriptive statistics for the participants included in further analyses.

**Fig. 2:**
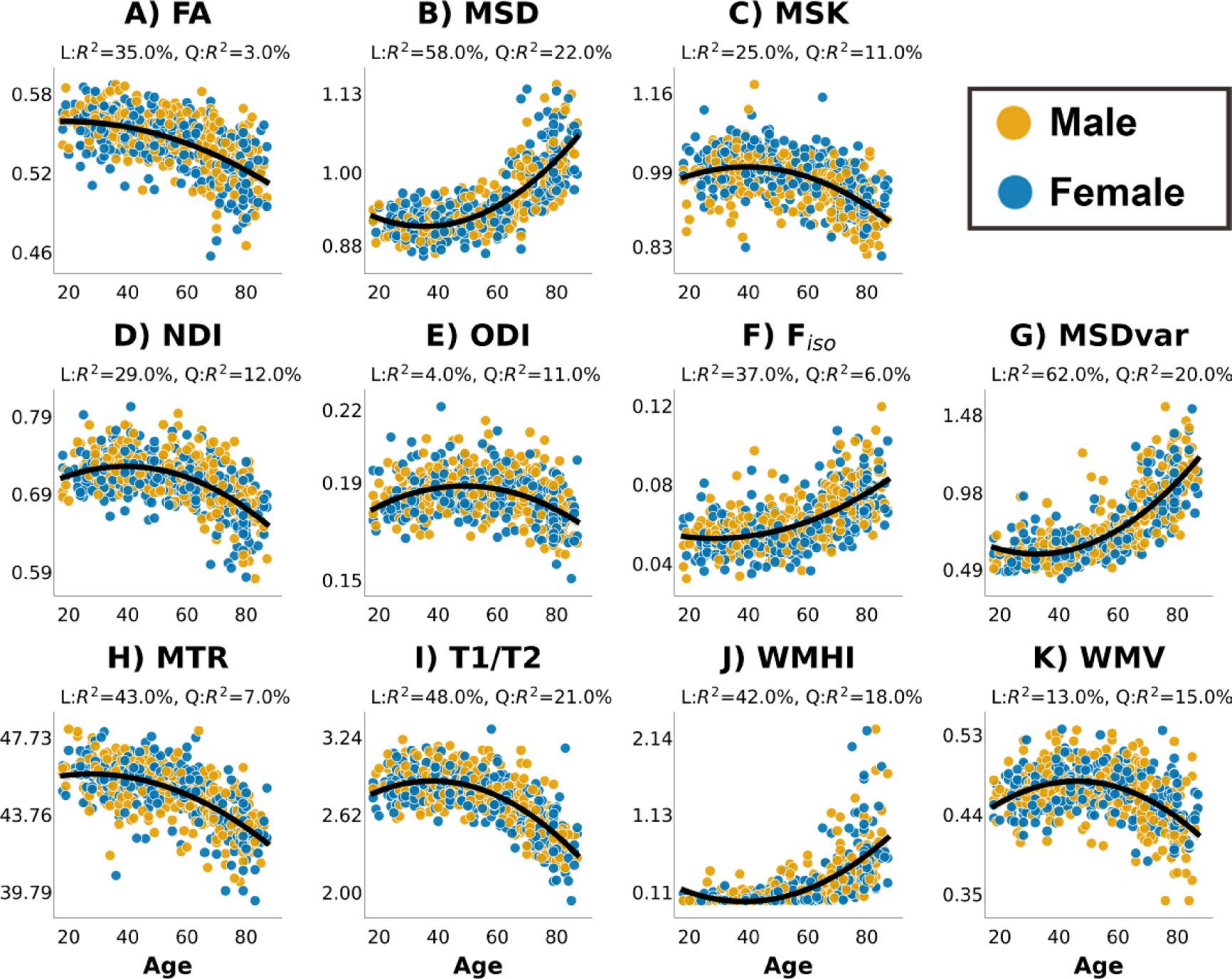
Values for all 11 WM metrics by sex and across age after removing outliers. (**A**) Fractional Anisotropy (FA); (**B**) Mean Signal Diffusion (MSD); (**C**) Mean Signal Kurtosis (MSK); (**D**) Neurite Density Index (NDI) from NODDI; (**E**) Orientation Dispersion Index (ODI) from NODDI; (**F**) Volume Fraction of Free isotropic water (F_iso_) from NODDI; (**G**) MSDvar - range of MSD values across 27 WM tracks; (**H**) MTR values across age; (**I**) T1w vs. T2w ratio; (**J**) Volume of White Matter Hyper-Intensities (WMHI) normalised by head size. (**K**) total White Matter Volume (WMV) volume corrected for head size. The proportion of variance (R^2^ explained by the Linear (**A**) and Quadratic (Q) effects of age (after accounting for main effect of sex and sex-by-age interactions) is shown at the top of each panel.

At least some type of valid MR data was available on total of 708 participants. However, for our first question of investigating the number of latent factors (and relative importance of different WM measures (see Fig. 1), including whether non-DWI sequences add complementary information), we used only participants who had data on all of the MR sequences, to avoid biasing the decomposition method towards the T1w and T2w sequences (which were the most common across participants). A total of 583 participants had valid data for all four MR sequences (T1w, T2w, MTw, DWI). We subsequently removed 13 participants who had outlier scores on at least one of the 11 WM measures described below, leading to a final sample of *n* = 570 (see Fig. 2). Outliers were defined as having residual values more than 5 SDs from the mean, after accounting for second order age and sex effects.

For the second question, where we related WM factors to cardiovascular health and to cognition, we maximised our sample size by including all *N* = 708 participants for whom we had any WM, cardiovascular, or cognitive measure, and used Full-Information Maximum Likelihood (FiML) methods to impute missing data^94–96^. Outlier values were also treated as missing data. For completeness, there were *n* = 607 participants with DWI measures; *n* = 584 with MTR values; and *n* = 640 with metrics derived from T1w and T2w sequences (TWM, WMHI and T1/T2).

### Cardiovascular measures

For cardiovascular measures, we used blood pressure (BP, *n* = 579), pulse pressure (PP, *n* = 579), heart rate (HR, *n* = 580), heart rate variability after low- and high-pass filtering (HRV_LF/HF, *n* = 604) and body-mass index (BMI, *n* = 587).

### Cognitive measures

For cognitive measures, we chose the fluid abilities of fluid intelligence (*n* = 660), processing speed (*n* = 658) and episodic memory (*n* = 706). Fluid intelligence was measured using the Cattell Culture Fair test, which included 4 sub tests^97^. We performed factor analysis to reduce the four sub-tests into one factor. Processing speed was captured across two tasks - simple and choice reaction time (RT) tasks – both of which involved one of four lights triggering one of four finger presses (full details in Shafto et al., 2014). The mean and standard deviation of RTs across 40–60 correct trials were computed for each task. To account for the fact that both mean and standard deviation of RTs were positively skewed, we inverted RTs before computing mean and standard deviation^98–100^. These four measures were then standardised, and reduced to a single latent variable using factor analysis. Episodic memory was measured using immediate recall, delayed recall and delayed recognition memory scores from the Wechsler Logical Memory task (Wechsler, 1991), and factor analysis was again used to reduce these three measures into a single factor. For all factor analyses, missing data were imputed with Full Information Maximum Likelihood.

### MRI acquisition

All imaging data were collected on a Siemens Trio 3T MRI scanner with 32-channel head coil. T1w and T2w images had 1 mm isotropic voxel size, FOV = 256 × 240 × 192 mm and flip angle = 9°. The T1w was acquired with an MPRAGE sequence with TE of 2.99ms and TR of 2250ms; the T2w was acquired with a SPACE sequence with TE of 408ms and TR of 2800ms. Both were acquired with acceleration factor 2 using GRAPPA sequence.

MTw images were collected with voxel size of 1.6 mm isotropic, FOV = 192 × 192 × 192 mm, TE = 5ms, and flip angle = 12°, acquired using a spoiled gradient echo sequence with TR = 30ms or TR = 50ms, depending on whether the participant’s specific absorption rate (SAR) estimation for the TR = 30ms sequence exceeded the stimulation limits. Two images were acquired, with or without a Gaussian RF saturation pulse with an offset frequency of 1950 Hz (bandwidth = 375 Hz, 500° flip angle, duration = 9984 µs).

DW images were collected with voxel size of 2 mm isotropic, FOV = 192 × 192 × 132 mm, TE = 104ms, TR = 9100ms, partial Fourier of 7/8, acceleration factor of 2 using GRAPPA with 36 reference slices, acquired using echo-planar imaging with twice refocused spin echo (TRSE) to reduce eddy-current artefacts. Diffusion sensing gradients were applied along 30 non-collinear directions for each of the two b-values (b = 1000 and b = 2000 s/mm^2^), together with three acquisitions without diffusion weighting (b = 0 s/mm^2^). More information about the MRI acquisitions and QC for the structural data is reported in Taylor et al. (2017), while full sequence parameters are here: https://camcan-archive.mrc-cbu.cam.ac.uk/dataaccess/pdfs/CAMCAN700_MR_params.pdf.

### Image pre-processing

The T1w and T2w images were initially processed using the SPM12 software (Wellcome Centre for Human Neuroimaging; https://www.fil.ion.ucl.ac.uk/spm), release 4537, implemented in the Automatic Analysis (AA) pipeline, release 4.2 (Cusack et al., 2015). Pre-processing is described in Taylor et al.^101^but in brief, the T2w image was rigidly coregistered to the T1w image, and then both were registered to a Montreal Neurological Institute (MNI) template brain (just to improve starting point for segmentation). Visual inspection checks were performed to ensure co-registration of T1w and T2w images as well as accurate normalisation and segmentation of the structural images^101^. Visual checks were also performed to ensure participants had no stroke related brain pathology and did not move during the magnetisation transfer sequences. Both images were corrected for B1 bias using the combined segmentation approach as implemented in SPM’s multimodal, unified segmentation and normalisation^102^. We computed total white matter volume based on the native space tissue probability maps calculated during multi-modal segmentation pipeline using T1w and T2w images as implemented in SPM 12. Total intra-cranial volume (TIV) was used to normalise the volumetric white matter measures.

As part of the AA pre-processing, we used the DARTEL algorithm^103^ to create a mean gray-matter template across the whole sample of participants, which was subsequently 12-parameter affine transformed to MNI space. The inverse of these combined transformations was then applied to convert the John Hopkins University DTI atlas JHU^104^ regions of interest (ROIs) back into the native space image of each participant’s T1w/T2w and MTR images.

The raw T1w and T2w images were also processed with the MRTool within SPM12^31^. The T2w image was coregistered to the T1w image using a rigid-body transformation. A transmit field intensity inhomogeneity (B1 field) correction was then applied to each image separately in native space, since they might have different intensity non-uniformity^105^. A new image was then created by dividing the T1w value by the T2w value at each voxel.

The two MTw weighted images were rigid-body coregistered and used to calculate the magnetisation transfer ratio (MTR) as (M0 - Ms)/M0, where Ms is the mean signal intensity with the saturation pulse and M0 is that without saturation.

The DWI data were pre-processed with a common pipeline that has been described in detail in Winzeck^106^. Pipeline included visual and semi-automated quality checks, including measures of signal to noise ratio, motion, brain extraction and detecting physiologically implausible values. Diffusion Imaging in Python DIPY (version 0.15.0 https://dipy.org/) procedures were implemented to (1) reduce noise via Principal Component analysis (PCA)^107^ and (2) correct Gibbs artefacts using an adapted sub-voxel shift procedure^108,109^. Eddy current distortions and head movement were corrected using eddy in FSL (https://fsl.fmrib.ox.ac.uk/fsl/fslwiki/). Correction for B0 inhomogeneity was not applied as reverse phase-encode direction data were not available.

Six DWI metrics for each voxel were computed as described in Henriques et al.^72^: (1) Fractional Anisotropy (FA) computed from standard diffusion tensor imaging (DTI); (2) Mean Signal Diffusion (MSD) and (3) Mean Signal Kurtosis (MSK) from the Mean Signal Diffusional Kurtosis Imaging (DKI) as fitted in the DIPY package^54^; and (4) Neurite Density Index (NDI), (5) Orientation Dispersion Index (ODI), and (6) isotropic Free water volume fraction (F_iso_) estimated from Neurite Orientation Dispersion and Density Imaging (NODDI) modelling. In brief we computed FA from fitting DTI model that excluded the b = 2000 s/mm^2^ values, using weighted linear least square fitting in FSL. Mean diffusivity and Mean kurtosis were calculated from kurtosis fitting as implemented in the DIPY package, and were calculated from extracting the average signal across different gradient directions see^54,55,110^. The NODDI model was fitted using the MDT toolbox (https://mdt-toolbox.readthedocs.io/en/latest_release/mle_fitting.html). Some illustrative images of these 6 diffusion metrics, and the MTR and T1/T2 estimates, are shown in Fig. 1.

### Region of interest (ROI) definition

WM tract ROIs were defined by the John Hopkins University (JHU) atlas^104^. WM metrics were obtained by first averaging across all voxels within the 48 WM tracts, and then averaged again over homotopic ROIs to create 27 WM ROIs. For the DWI data, the ROIs were back-projected from an FA template into each person’s FA native space using FSL’s linear and non-linear registration tools^111–113^. To suppress the impact of free-water partial volume effects from cerebral spinal fluid (CSF), and to minimise the impact of degenerative ODI and NDI estimates in voxels containing mostly free water, voxels with F_iso_ values larger than 0.9 were removed from the ROIs. For the T1/T2 and MTR data, we used the same ROIs that were inverted back to native space using the inverse DARTEL warps derived as a part of the AA pipeline described above.

To match the whole-brain metrics below, the WM metrics were averaged across the 27 ROI tracts to create a single global measure of white matter integrity from each measure. In supplementary materials, we show factor analysis performed after concatenating each of the 11 measures across all 27 ROIs (see Supplementary Figs. 1, 2 and 3).

### Whole-brain metrics

We extracted total white matter (WMV) volume from SPM 12’s segmentation procedure. We then regressed out total intracranial volume (TIV), also estimated by SPM12, to ensure the measure was not confounded by head size.

While WMHIs are usually measured by a FLAIR (Fluid-Attenuated Inversion Recovery) sequence, recent developments of the SAMSEG algorithm allow them to also be estimated from T1w and T2w volumes^114^. The SAMSEG algorithm is a multi-atlas probabilistic segmentation method that has been shown to be robust to noise and the inherent intensity inhomogeneity in the MRI data. For each voxel, the SAMSEG algorithm outputs a probability that it contains a white matter hyper-intensity lesion. We used SAMSEG as implemented from Freesurfer version 7.4.0 (https://surfer.nmr.mgh.harvard.edu/fswiki/Samseg)^116^. We used a probability threshold of 0.5 to define binary lesion masks across the whole brain, from which we computed WMHI lesion volumes. We then normalized this WMHI volume by TIV, like for WMV above.

The last measure represented the range of values across voxels present in the mean signal diffusion (MSD) image, and is conceptually identical to the recently proposed measure of “peak width of skeletonised mean diffusivity” (PSMD) (Baykara et al., 2016). The main difference here was that we computed the 90% percentile range of MSD values for all voxels within the 27 ROI tracts, rather than within an FA skeleton. This “MSDvar” measure was highly correlated with the standard PSMD measure (*r* = 0.86) taken from King et al.^90^.

### Statistical analysis

We had two main aims in this manuscript. The first one was to summarise the relationship between different WM measures. Specifically, we wanted to examine which WM measures are strongly interrelated with others and whether non-DWI measures provide complementary information not captured by DWI metrics. The second main question was to examine how WM health relates to cognition and Cardiovascular measures. The derived white matter measures are available as csv files hosted on OSF. We also provide R code to reproduce the main analyses https://osf.io/y7ct8/. Below we describe the approaches for the 2 main questions in the paper. To examine how white matter relates to cognition and cardiovascular health we ran two structural equation models (SEMs). When testing the significance of the SEM paths, we did not correct for multiple comparisons across all the paths, because each path is dependent on the specific structure of the whole model.

1. Using Principal Component Analysis to test relationships between WM measures

Principal Component analysis (PCA) was applied to all 11 WM measures, after Z-scoring each measure separately. We used recently developed, element-wise cross-validation to identify the number of components, as implemented in the MEDA Matlab toolbox^116,117^. This element-wise k-fold cross-validation (*ekf*) has been shown to be more appropriate than the standard whole observation (holding out whole row) cross-validation for PCA^117–121^. Note however that ekf is only one of many ways to estimate number of components, and each method seems to be differentially sensitive to different aspects to the data^121^. After determining the number of components, we used the “Varimax” rotation to enhance interpretability.

2. Using path models to validate WM factors against cardiovascular health and cognition.

Here we attempted to validate the above WM factors by relating them to independent physiological and cognitive data. We ran two separate path analyses, using the lavaan R package^96^. In one path model, we examined how the WM factors were predicted by latent cardiovascular factors, in line with the idea that cardiovascular problems lead to white matter damage. In the second path model, we examined how the WM factors predicted the cognitive measures of fluid intelligence, processing speed and episodic memory. In other words, the two path models allowed us to examine both the potential causes (cardiovascular) and consequences (cognitive) of the WM factors. In both of these path models we show paths with and without controlling for linear and quadratic age effects and sex. Additionally, based on a reviewer suggestion, we ran multi-group SEM models to test whether the paths between latent factors varied with age, in both the cardiovascular and cognitive SEM models. These models are reported in supplementary results, but in brief we found no evidence that the relationships reported in the manuscript were moderated by age (see Supplementary Materials).

Note that some cohorts may not have, or be able to acquire, all the WM metrics utilised here. Therefore in Supplementary materials, we also report how each individual WM measure was independently related to cognition, so that researchers might be able to choose the ‘best’ metric (and MR sequence) if their goal is to relate WM to cognition (see Supplementary Figs. 4 & 5).

### Covariates

We report the two path models both with and without including polynomial age and sex effects on the outcome variable in the same model. Regressing out age effects can remove some of the variance in WM factors caused by age-related but non-cardiovascular variables, and some of the variance in cognition caused by age-related but non-WM variables. However, age is also likely to cause individual differences in cardiovascular and/or WM, so the danger of regressing it is that true cardiovascular-WM and/or WM-cognition relationships are masked. This is a limitation of cross-sectional data^122–124^but such data can at least be used to identify potential links between these variables that can be further tested in longitudinal data.

Another source of individual differences in brain and cognition is genetics. A large proportion of the between-person variance in intelligence is attributable to early-life differences in intelligence^125^. Given that intelligence is highly heritable^126^we also adjusted for potential genetic effects in the second path model in which WM predicted cognition. Prior work in Cam-CAN has examined single gene effects^127,128^but here we used a polygenic score (PGS) for general cognitive ability with the single nucleotide polymorphisms (SNP) effect sizes identified in a GWAS study from other independent cohorts^129^. The PGS was computed, independently of our other cognitive and brain measures, using the Bayesian continuous shrinkage prior approach, which robustly infers the posterior effect sizes of SNPs, while also taking into account the external linkage disequilibrium^130^. PGS was adjusted for the first 10 genetic principal components (to control for population stratification effects). Because the adjusted PGS scores are on an arbitrary scale, they were z-scored across participants. This standardisation means that an individual with a PGS score of 2 has a genetic risk for a particular trait that is about 2 SD above the average risk of the sample.

## Results

We first explored each of the 11 WM measures in terms of the relationship with the age (linear and quadratic) and sex of each participant (Fig. 2). The results of the full regressions are in Supplementary Table 2. We briefly summarise them here. The plot shows how the 11 WM measures differ across age after accounting for sex and sex-by-age interactions. The individual associations between age and white matter measures were not corrected for multiple-comparisons as they were not the main focus of the paper. We note however that only two effects would not survive a Bonferroni-correction (the main sex effect on T1/T2 and the age-by-sex interaction on WMHI).

Most measures exhibited a main effect of sex, except for MSD, ODI, MTR, WMHI and WMV. The measure with largest sex difference was MSK, where females tended to have higher values (β = 0.007, t = 3.80 *p* < 0.001, η^2^ = 0.025), though this effect size was still relatively small.

All measures showed a large, main effect of age (see Fig. 2 for effect sizes for Linear and Quadratic components). The results for the 6 DWI measures were qualitatively very similar to those reported by Henriques et al.^72^so we focus on the 5 new measures. The “MSDvar” DWI metric tended to increase quadratically with age, similar to the mean MSD values. Indeed, both of these MSD metrics showed the strongest effect of age (with linear and quadratic components combining to explain 79% and 82% of their variance respectively). On the other hand, MTR and T1/T2 ratio decreased quadratically with age. There was a strong non-linear increase in WMHIs with age, which were most prominent in old age, in line with prior work^7,18,70,88,131–134^. WMV exhibited an inverted U-shape relationship across the whole age range.

### Principal component analysis for the 11 WM metrics

**Fig. 3:**
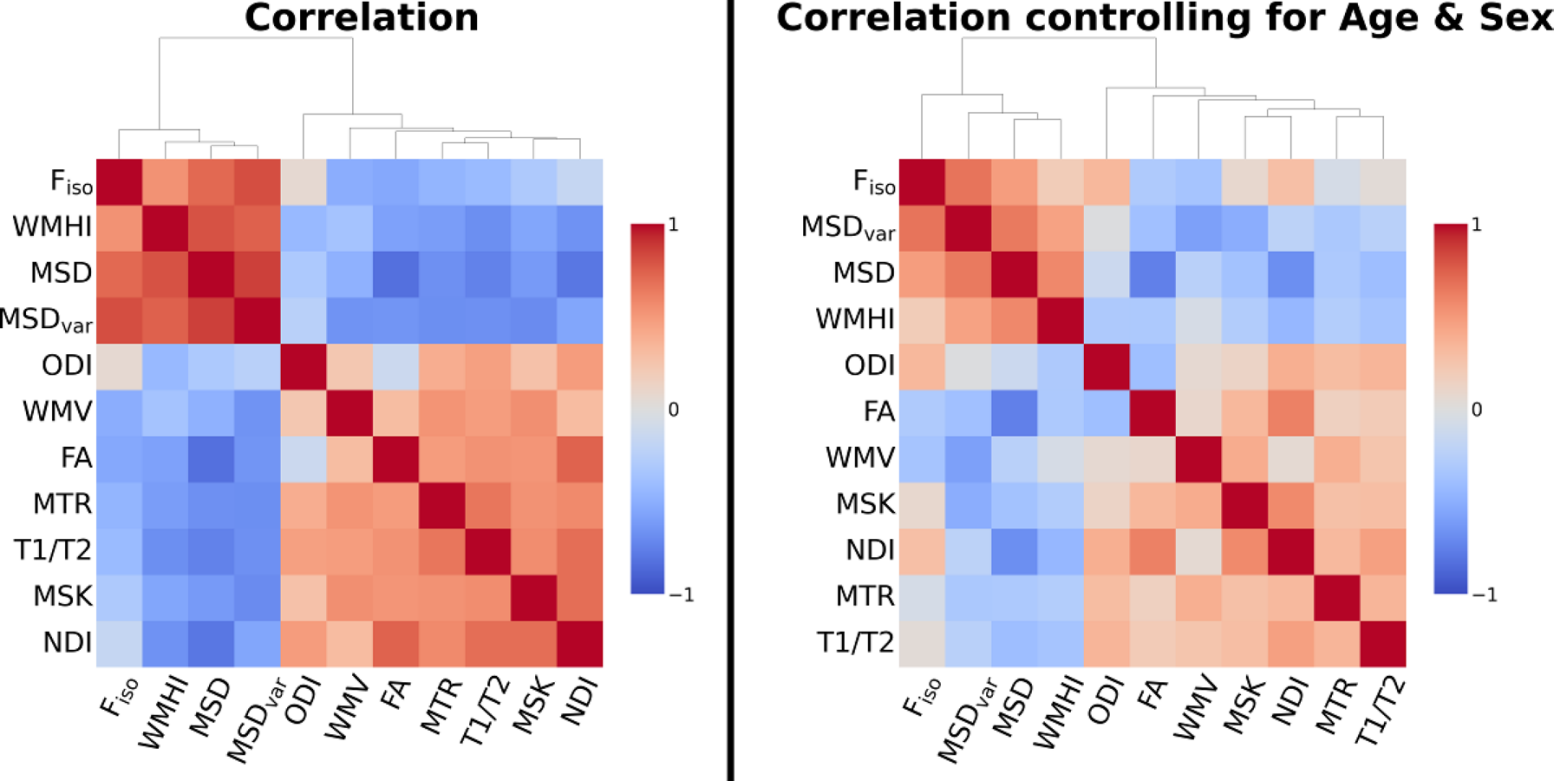
Correlation matrix for all 11 WM measures across participants before (left) and after (right) correcting for sex, linear and quadratic age effects, and the sex-by-age interactions. Metrics are ordered according to the dendrogram of agglomerative similarity between measures.

Figure 3 shows the correlation among the 11 WM measures, before (left) and after (right) adjusting for second-order effects of age, sex and their interactions. We first regressed out the confound variables and then computed correlation of the residuals. We note this is more aggressive clean-up as likely there is variance shared across white matter measures and age. The high similarity between some measures even after partialing effects of age indicates that the relationships between metrics are not driven solely by common age dependency. In particular, high positive correlations are observed among F_iso_, WMHI, MSD and MSDvar, and between FA, WMV, MTR, T1/T2, MSK and NDI.

We used ekf cross-validation to determine the number of PCA components. We note that this element-wise cross-validation method has recently been suggested as a robust way to determine number of PCA components^117,119^. To ensure our factors were interpretable we performed Varimax rotation on the 4 components.

**Fig. 4:**
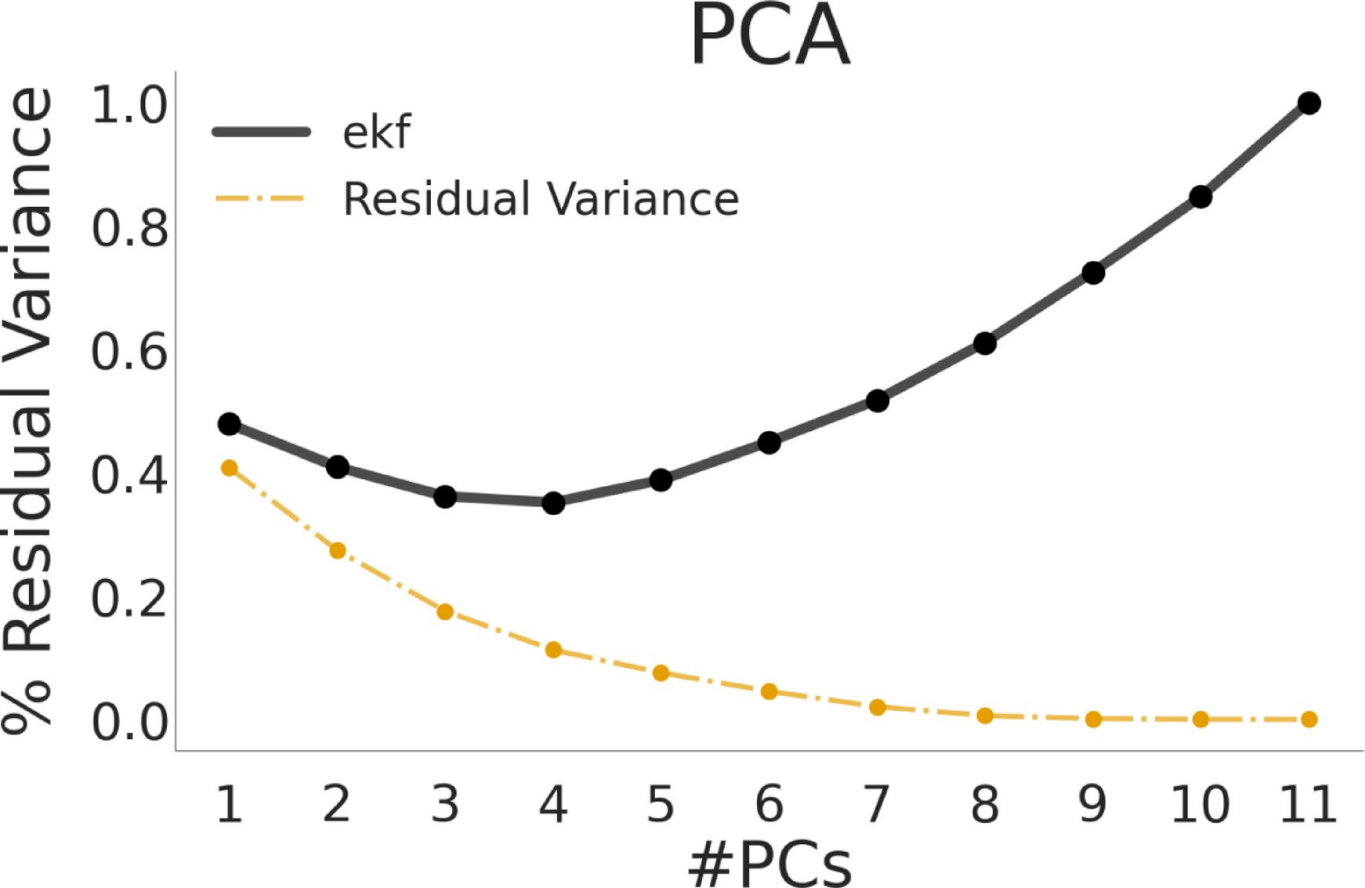
PCA cross-validation. The figure shows the ekf cross-validated error as a function of number of principal components retained, along with the residual variance (inverse of variance explained) in orange. Based on the ekf, we selected 4 PCs and performed factor analysis with 4 factors.

The PCA with 4 components captured 88.7% of the variance in the 11 WM measures. The 1st principal component captured 59.2% of the variance, the 2nd PC explained 13.4%, the 3rd explained 9.9% and the 4th component explained 6.3% of the variance. The 5th component that was not retained captured 3.7% of the variance (Fig. 4).

**Fig. 5:**
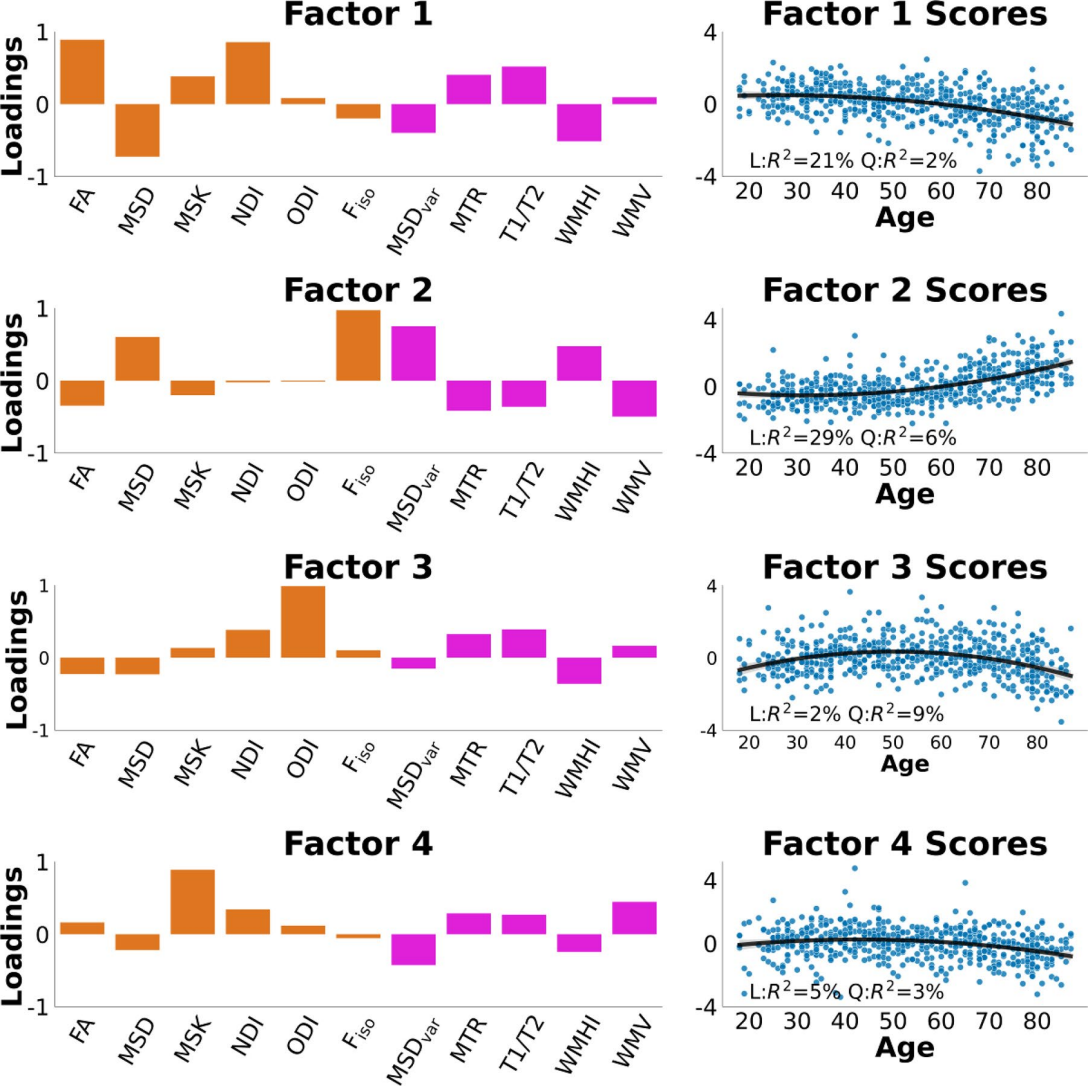
Loadings and Factor scores across age. We show factor loadings across all 11 WM measures and how the factor scores varied with age. We interpret Factor 1 (WMF1) as a microstructural properties/myelination factor. Factor 2 (WMF2) as free-water/tissue damage factor. Factor 3 (WMF3) as fibre-crossing complexity factor. Factor 4 (WMF4) as microstructural complexity.

Figure 5 shows the loadings for each factor and how the factor scores related to age. Overall, Factors 1, 2 and 3 resembled the factors found in Henriques et al.^72^. The first factor loaded strongly on FA and NDI, with a negative loading on MSD. This factor declined with age and likely reflects microstructural properties such as axonal fibre density and myelination. Interestingly, Henriques et al.^72^ also found that MSK loaded on this factor, whereas here it loaded more on the additional, fourth factor (see below). Factor 1 (WMF1) also loaded positively on MTR and T1/T2 ratio, consistent with age-related reductions in myelin content, and negatively on WMHI, consistent with demyelination with age.

Factor 2 (WMF2) loaded positively mainly on F_iso_ and MSD, most likely representing increases in free water content. This factor increased with age. Interestingly, MSDvar – the DWI measure conceptually identical to PSMD - also loaded positively on this factor, as did WMHI. Conversely, MTR, T1/T2 ratio and WMV all loaded negatively on this factor to some extent. This suggests that Factor 2 captures WM damage/lesions leading to increase partial volume effects between tissue and CSF.

Factor 3 (WMF3) mainly loaded positively on the ODI metric, with weak loadings on all other metrics, and showed a weak, inverted-U shape function of age. Like in Henriques et al.^72^this factor seems to relate to complexity of fibre configurations (such as crossing fibres and fibre dispersion).

Factor 4 (WMF4), which was not needed in Henriques et al.’s analysis of DWI metrics alone, loaded positively on MSK, with a smaller positive contribution from WMV and a smaller negative contribution from MSDvar. It showed a weak decline with age. Interpretation of this factor is less clear but appears to reflect general microstructural complexity/volume that is distinct from fibre density/myelination and free-water/lesions^134^.

### Validating the white matter factors against cardiovascular health and cognition

#### Cardiovascular measures predicting white matter

First, we examined whether the latent vascular scores predicted the latent WM scores. We defined 3 cardiovascular factors based on prior work from the Cam-CAN cohort^89^. The first latent vascular factor (LVF1) captured static blood pressure and related to BMI; the second (LVF2) captured pulse pressure and heart rate; the third (LVF3) loaded highly on both high and low frequency heart rate variability. We tested how these cardiovascular factors predicted the four WM factors (Supplementary Fig. 6). We had data from 627 participants. In Supplementary Fig. 7, we show the correlation matrix between age, white matter and cardiovascular factors, and cognitive measures.

**Fig. 6:**
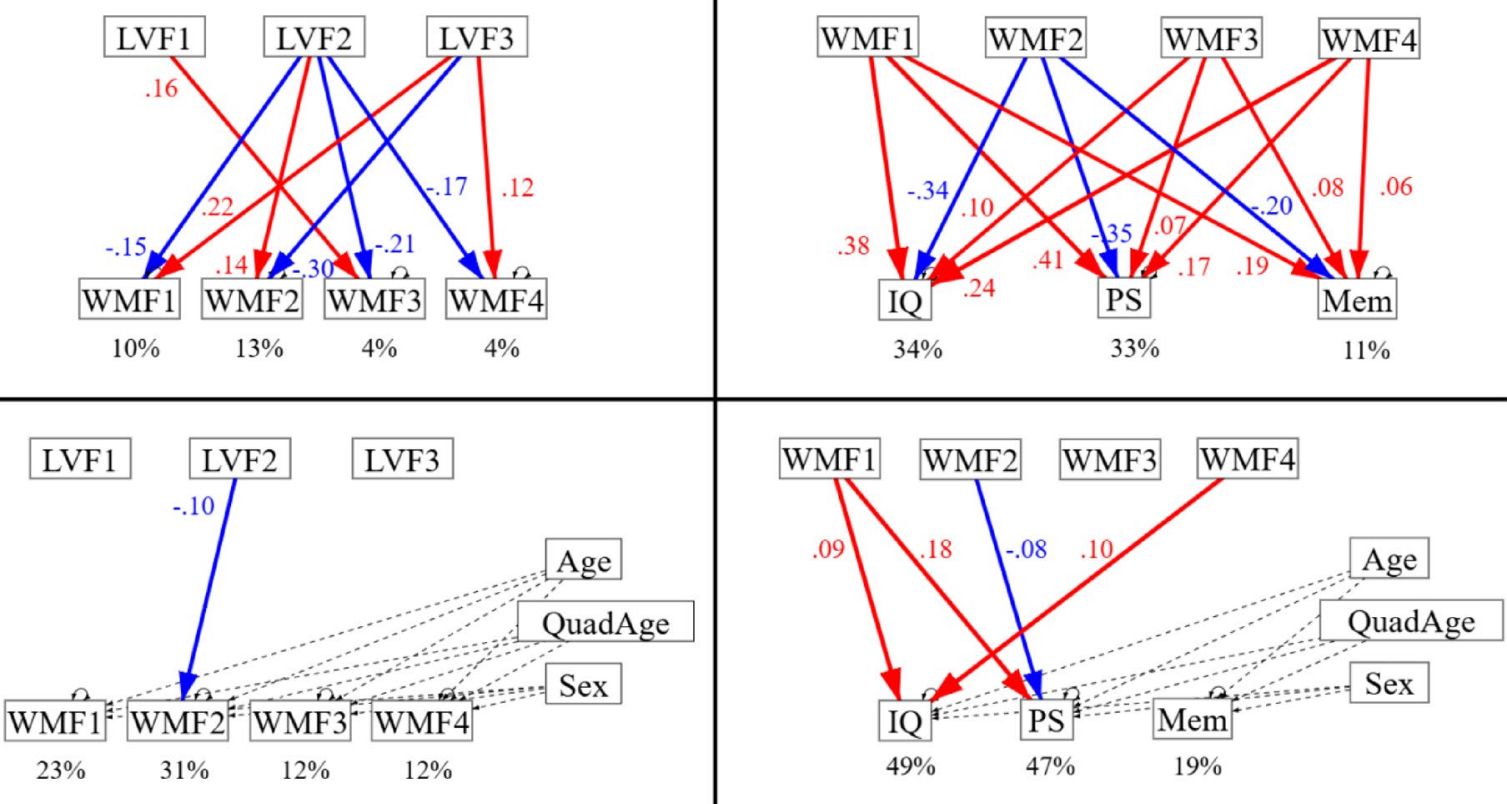
Path models relating cardiovascular factors to WM factors (left panels) and WM factors to Cognitive factors (right panels), without (top panels) and with (bottom panels) adjustment of outcome variables for Age and Sex effects. Solid coloured lines indicate significant paths. R-square values are shown as percentages below each dependent variable. Age and Sex were included as confounds of no interest, represented by dashed lines. LVF = latent vascular factor; WMF = white matter factor; IQ = fluid intelligence; PS = processing speed; Mem = episodic memory.

We first report the path model relating cardiovascular and WM factors (WMFs) without adjusting the WMFs for age and sex effects (Fig. 6, top left; see Supplementary Table 3 for full parameters^136^. This model would be appropriate if the effects of age and sex on WM were via their effects on cardiovascular health. This model showed WMF1 (reflecting fibre density/myelination) was negatively related to LVF2 (largely pulse pressure) but positively related to LVF3 (heart-rate variability). This is consistent with low pulse pressure and high heart-rate variability being associated with better cardiovascular health.

WMF2 (reflecting free-water content / WM damage) showed the opposite pattern of a negative path from LVF2 and positive path from LVF3, this time generally consistent with poor vascular health being associated with more WM damage. WMF3 (reflecting fibre-configuration complexity) was positively related to LVF1 (largely static blood pressure) and negatively related to LVF2 (pulse pressure). The reason for these is less clear. Finally, WMF4 was negatively related to LVF2 and positively related to LVF2, like WMF1.

We also report results from a model where (linear and quadratic) effects of age and sex affected WM through routes that are independent of cardiovascular heath (Fig. 6, bottom left). Because age in particular has such strong effects on WM, the only path from cardiovascular factors that remained significant was the path from LVF2 to WMF2, though this was now negative rather than positive (see Discussion). The paths to WMF3 from LVF1 and LVF2 also approached significance (Supplementary Table 4).

### White matter predicting cognition

The cognitive factors of fluid intelligence (IQ), processing speed (PS) and episodic memory (Mem) were based on theoretical distinctions rather than data-driven factor analysis (though each factor was itself estimated from factor analysis of several component scores; see Methods). Without accounting for age and sex, all WM factors were related to all cognitive factors (Fig. 6, top right; Supplementary Table 5). As expected, WMF2 (reflecting free-water content / WM damage) was negatively related to all three cognitive factors, whereas the remaining three WM factors were positively related to all cognitive factors, consistent with good WM health being associated with better cognitive outcomes. Nonetheless, these relationships could all be driven by independent effects of age on both WM and Cognition.

In the path model that allowed for such independent effects of age (and sex) (Fig. 6, bottom right and Supplementary Table 6), the positive effects of WMF1 on fluid intelligence and processing speed remained significant. The positive effect of WMF4 on fluid intelligence also remained significant. Finally, the negative effect of WMF2 on processing speed remained significant. Thus at least some contributions of WM to cognitive abilities appear independent of age, at least for fluid intelligence and processing speed. We did not include Education as a further covariate, because the direction of causality is unclear, given that education could lead to better cognition, but also individuals with higher cognitive abilities could be likely to spend longer in education in the first place^137–139^. Nonetheless, based on a reviewer suggestion, the results from additionally controlling for Education are shown in Supplementary Table 7. The pattern of significant results was largely unchanged; the only difference was that WMF2 now also became significantly associated with fluid intelligence.

### White matter predicting cognition controlling for polygenic effects

Finally, Cam-CAN cohort also has a polygenic score (PGS) for general cognitive ability. We therefore ran a final path model in which the cognitive outcome variables were also adjusted for this PGS. As expected, this PGS was positively associated with all our cognitive measures (Supplementary Tables 8 and Supplementary Fig. 8). This score is only available in a sub-sample of *n* = 565 participants, but due to missingness in both neuroimaging and cognitive data, the final path model included only 505 participants.

**Fig. 7:**
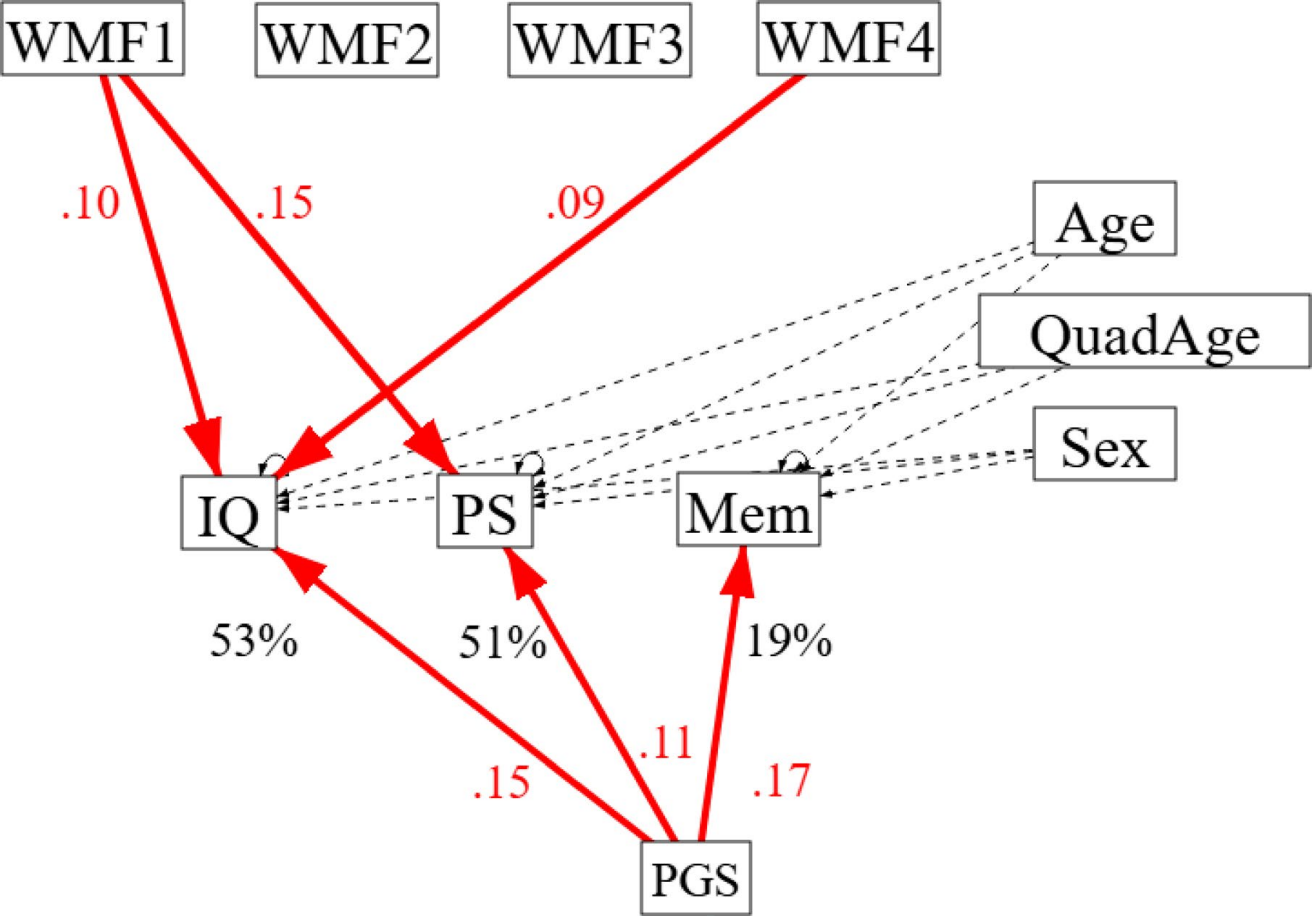
Path Model for effects of WM on Cognition, now additionally controlling for polygenic scores (PGS) for cognitive ability.

The positive paths between WMF1 and WMF4 and fluid intelligence and processing speed remained significant even when controlling for this genetic contribution (Fig. 7 and Supplementary Table 8), though the negative path from WMF2 to processing speed no longer reached significance (though this could also reflect the lower power from this reduced subset of participants). Interestingly, the PGS was not significantly correlated with any of the WM factors (Supplementary Table 8), consistent with the associations between WM and cognition being at least partly due to environmental and lifestyle factors.

## Discussion

Using a large sample of healthy adults, we examined the relationship between 11 different white matter (WM) metrics, derived from 4 different MR contrasts / sequences (T1w, T2w, MTR and DWI), their relationships with age and sex, their underlying factor structure, and the relationship of those factors with cardiovascular health and cognitive abilities. Cross-validated PCA suggested that there are 4 WM factors, which captured 89% of the variance in the 11 metrics. After Varimax rotation, these factors seemed to represent fibre density / myelination (WMF1), free water / WM damage (WMF2), crossing-fibre complexity (WMF3) and general microstructural complexity / volume (WMF4). Path models showed that many of these factors were affected by 3 latent factors derived from 6 measures of cardiovascular health, at least one of which (the negative effect on WMF2 of a vascular factor related to pulse pressure) was independent of any shared effects of age or sex. Moreover, many of these factors predicted the cognitive abilities of fluid intelligence, processing speed and episodic memory, even after adjusting for age and sex, at least in the case of fluid intelligence and processing speed. Indeed, the positive effects of WMF1 and WMF4 on fluid intelligence remained even after further adjusting for polygenic disposition for high cognitive ability, consistent with environment/lifestyle contributions.

Our PCA results highlight that multiple potential WM properties underlie common MR contrasts, consistent with previous studies^70,138^. This suggests that a single MR metric is insufficient to capture fully the effects of variables like age and cardiovascular health on WM. Our previous study identified 3 components underlying 6 metrics from diffusion-weighted MRI^72^. Here, the addition of a further 5 metrics, 4 of which were from 3 new MR contrasts, resulted in the need for a fourth component, suggesting some complementary information in T1w, T2w and MTR sequences. However, the fourth component only explained an additional 6.3% of variance in the 11 WM metrics, and other methods for identifying the optimal number of principal components may suggest fewer than four (there is no universally agreed method for deciding the number of factors). Indeed, if three components are in fact sufficient, then one could argue that DWI data alone are sufficient to capture of all these (when used to derive multiple diffusion-related measures, as here), and that there is no additional benefit of acquiring T1w, T2w and MTR data (Supplementary Table 9). In fact, the first three factors here seemed to correspond largely to the three metrics derived from the NODDI model of DWI data.

Nonetheless, even if three components are sufficient, the addition of the 5 extra WM metrics in the present paper helped bolster the interpretation of the WM factors that resulted. Furthermore, extracting latent factors from more WM metrics should in theory improve the accuracy (signal-to-noise ratio) of those factor loadings, by virtue of greater averaging over independent noise sources associated with each metric. Conversely, if one had to choose just one WM metric (from one MR contrast), then Supplementary Fig. 4 shows that MSDvar is likely to be most sensitive to age, MSK is likely to be most sensitive to fluid intelligence, FA is likely to be most sensitive to processing speed, and MTR is likely to be most sensitive to episodic memory (adjusting for age and sex in the latter three cases).

We only considered “global” measures, averaged across 27 ROIs of the JHU atlas. This was because some of the common WM metrics that we wanted to include, such as PSMD are by their nature only defined across multiple WM regions/voxels (here, defined in terms of the 5–95% range of MSD values across ROIs). Nonetheless, in Supplementary Material, we applied PCA after vertically concatenating the 27 ROIs for each participant. The PCA ekf cross-validation again suggested 4 factors, which showed very similar factor loadings to the global approach and factor scores that were highly correlated with the global ones. Thus, while we are not arguing that there is no important regional variation in white matter, it is noteworthy that the global factor analysis approach is sufficient to identify the principal factors of white matter variation. This may be beneficial to reduce the number of comparisons when testing associations between lifestyle, genetics and cognition. However, certain WM metrics might be better suited for certain parts of the brain, which could be the topic of future research.

We sought external validation of the four WM factors by relating them to potential cardiovascular causes. More specifically, we identified 3 vascular factors (LVFs) from 6 measures in CamCAN (systolic and diastolic blood pressure, body-mass index, heart rate, and heart-rate variability after low- and high-pass filtering of ECG), based on prior work by^89^. Significant relationships were found between most of the LVFs and most of the WMFs, providing convergent construct validity for the WMFs.

When we adjusted the WMFs for linear and quadratic age effects, many of the contributions from LVFs were no longer significant. With cross-sectional data as here, it is difficult to determine whether the observed relationships between LVFs and WMFs are simply artefacts of independent correlations with age. Alternatively, the reduction in this relationship after adjusting for age can reflect the fact that age is the primary driver of vascular health^141^ with age-related variation in LVF subsequently causing variation in WM. It is likely that both of these scenarios are true to some extent. Nonetheless, at least one path survived adjustment for age: that from the LVF representing pulse pressure and the WMF capturing free water / WM damage. This is in line with prior work showing a relationship specifically between pulse pressure and WM health in middle-aged and older adults^76–79,88^. Note however that the path was negative, such that higher pulse pressure appeared protective. This was actually a flip in sign compared to when age was not adjusted for (when the relationship was positive, as expected). This suggests that age is a common cause of both increased pulse pressure and WM damage. We note that similar reversal of direction of effect when age is included was observed in previous cross-sectional work in the UK Biobank^80^highlighting the need for longitudinal and treatment studies to better understand the causal mechanisms through which pulse pressure is associated with WM damage.

Further convergent evidence for the validity of the WMFs was obtained when relating them to cognition. Even after adjusting for age and sex effects, we found that the WMF associated with fibre complexity / myelination and WMF associated with microstructural complexity / volume predicted unique variance in fluid intelligence. The WMF associated with fibre complexity / myelination also predicted processing speed, but this time there was also a unique (and negative) contribution from the WMF associated with free water / WM damage. The fact that the WMF associated with fibre complexity / myelination loaded on FA and NDI and MSD measures is consistent with previous studies that have found relationships between these individual metrics and fluid intelligence and/or processing speed in middle aged and older cohorts^3,9,64,69,98,142–149^. It is worth noting though that FA and MSD also loaded appreciably on other WMFs, suggesting that these individual metrics are likely to capture multiple different aspects of WM health.

Interestingly, the fourth WMF related to microstructural complexity / WM volume and which made a unique contribution to fluid intelligence, loaded mainly on mean signal kurtosis (MSK). Kurtosis imaging captures the non-Gaussian diffusivity in WM tracts, and lower MSK values are thought to reflect reduced tissue complexity, as they indicate water diffusion is more Gaussian or unrestricted by the presence of neuron axons, dendrites and cells. It potentially provides a broader measure of microstructural integrity than DTI measures or the information captured by NDI. This is consistent with the idea that age-related processes lead to changes in multiple cell types in WM tracts, and these changes are best captured by a broad measure like MSK^83^. It may therefore be a useful measure for future studies of neurocognitive ageing.

Beside our main focus on the latent WMFs, we also examined the relationship between each individual WM metric and cognition (see supplementary materials). T1w/T2w, WMV and WMHI all showed strong age-related changes, but these measures did not show robust relationship with cognition after accounting for age effects. While the T1w/T2w ratio has gained some popularity recently, these results suggest that it may not be a particularly useful measure to understand cognition. This is in line with recent work suggesting that the T1w/T2w ratio in WM tracts is not particularly sensitive to myelin content and may be difficult to interpret^32,44–46,150^. On the other hand, given the large number of neuroimaging studies reporting an association between WMHI, age and cognition^18,20,134,145,151,152^we were surprised to only see a weak association between WMHI and cognitive performance. However, there may be good reasons for this. First, we computed WMHI using T1w and T2w images, rather than the FLAIR images – which better capture the margins of white matter lesions. Second, we focused on a whole adult life-span sample, whereas most prior work on WMHI and cognition has focused on older adults, particularly those with cardiovascular disorders. Third, it may be important to consider the number and/or location of the WMHI lesions, rather than focusing on just their combined volume, as here^153,154^.

There are other limitations to this work that are important to consider. The cross-sectional nature of the data prevented us from testing how longitudinal changes in WM relate to age-related cognitive changes^5,123,124,155^. It is possible that different white matter measures are differentially sensitive to biological processes that are occur at different rates during aging. Secondly, we used linear latent variable methods such as PCA, but it is possible that there are non-linear associations between the different WM measures^154,155^. Thirdly, we did not consider regional variation in WM across the brain, for reasons explained above, nor did we have histological data to provide stronger evidence for interpreting the different white matter factors. Lastly, in order to use comparable brain regions across metrics, we used the same set of atlas-based major WM tracts, inverse-normalised into the native space of each participant; it might be possible to extract more sensitive metrics by aligning participants to a WM skeleton (based on DTI) defined in each individual’s native space (as done, for example, for standard PSMD^63^. We also removed voxels from ROIs whose Fiso value (estimated proportion of CSF) was greater than 0.9, which was necessary on order to estimate sensible NDI and ODI parameters, but the number of such excluded voxels could also vary with age, potentially influencing the effect of age.

In conclusion, we show that utilising multiple white matter measures from multiple MR contrasts, together with dimensionality reduction techniques, can lead to robust and interpretable white matter health measures. We show that 4 factors are sufficient to capture the majority of variance in those measures, and that these factors uniquely relate to cardiovascular and cognitive measures. We argue that this multidimensional approach to white matter health may be a fruitful avenue to better understand the links between aging, brain and cognition.

## Supplementary Information

Below is the link to the electronic supplementary material.

Supplementary Material 1

## Data Availability

The raw data are in BIDS format are available on request from this website: https://camcan-archive.mrc-cbu.cam.ac.uk/dataaccess/. The pre-processed ROI and cognitive data and code to reproduce the main results from the pre-registered analyses are available here - https://osf.io/y7ct8/.
