# Supplementary material for: Complementary MR measures of white matter and their relation to cardiovascular health and cognition
Source: Sci Rep. 2025 Aug 7;15:28890. doi: 10.1038/s41598-025-13610-2 (PMC12332191; doi:10.1038/s41598-025-13610-2)
Supplement: Supplementary file 1 — Supplementary Material 1 [file 41598_2025_13610_MOESM1_ESM.pdf]

Supplementary Materials

Supplementary Table 1 Descriptive values for our sample. We show mean and standard deviation or count and prevalence as percentage. For more details, see Shafto et al. (2012).

**Characteristic**	N = 708
**Age** (range, which was approx. uniform)	18-89
**Sex**	
Male, number (percentage)	349 (49%)
Female, number (percentage)	359 (51%)
**Education**	
None, number (percentage)	46 (6.5%)
GSCE/O-level, number (percentage)	96 (14%)
A-level, number (percentage)	138 (20%)
Degree, number (percentage)	426 (60%)
**BMI**, *mean (standard deviation)*	25.8 (4.6)
**BMI between 18.5 and 25**, *number (percentage)*	274 (47%)
**Systolic Blood pressure**, *mean (standard deviation)*	121 (17)
**Diastolic Blood pressure**, *mean (standard deviation)*	73 (10)
**Number (%) with 130+ Systolic or 80+ diastolic**	200 (28%)

Factor analysis concatenating measure per ROI

Although our main results are focused on measures calculated across all white matter ROIs, or at the whole brain level (total white matter volume), here we show results when performing factor analysis after concatenating all 27 JHU ROIs for each participant (as in Henriques et al., 2023, averaged over bilateral ROIs). To make results comparable between the global and ROI factor analyses, we focused on the 570 individuals used in the main analyses. Thus we performed PCA on a 570*27 by 11 matrix. As for the global analysis, we performed element wise cross-validation using the MEDA toolbox to estimate number of PCA components, and then performed factor analysis using Varimax rotation. We again found that 4 components were optimal to summarise the data, and that the factors loadings were qualitatively very similar to the factors observed in main paper (Sup. Fig. 5). In order to compare the factors scores from the two analyses approaches we created an average factors score by averaging over ROIs. This allowed us to show how the factors scores correlate among each other and change with age. We show correlations between the factor scores from both methods (Sup. Fig. 6). We also show ROI loadings to provide some intuition about regional variations for the factor scores (Sup. Fig. 7).

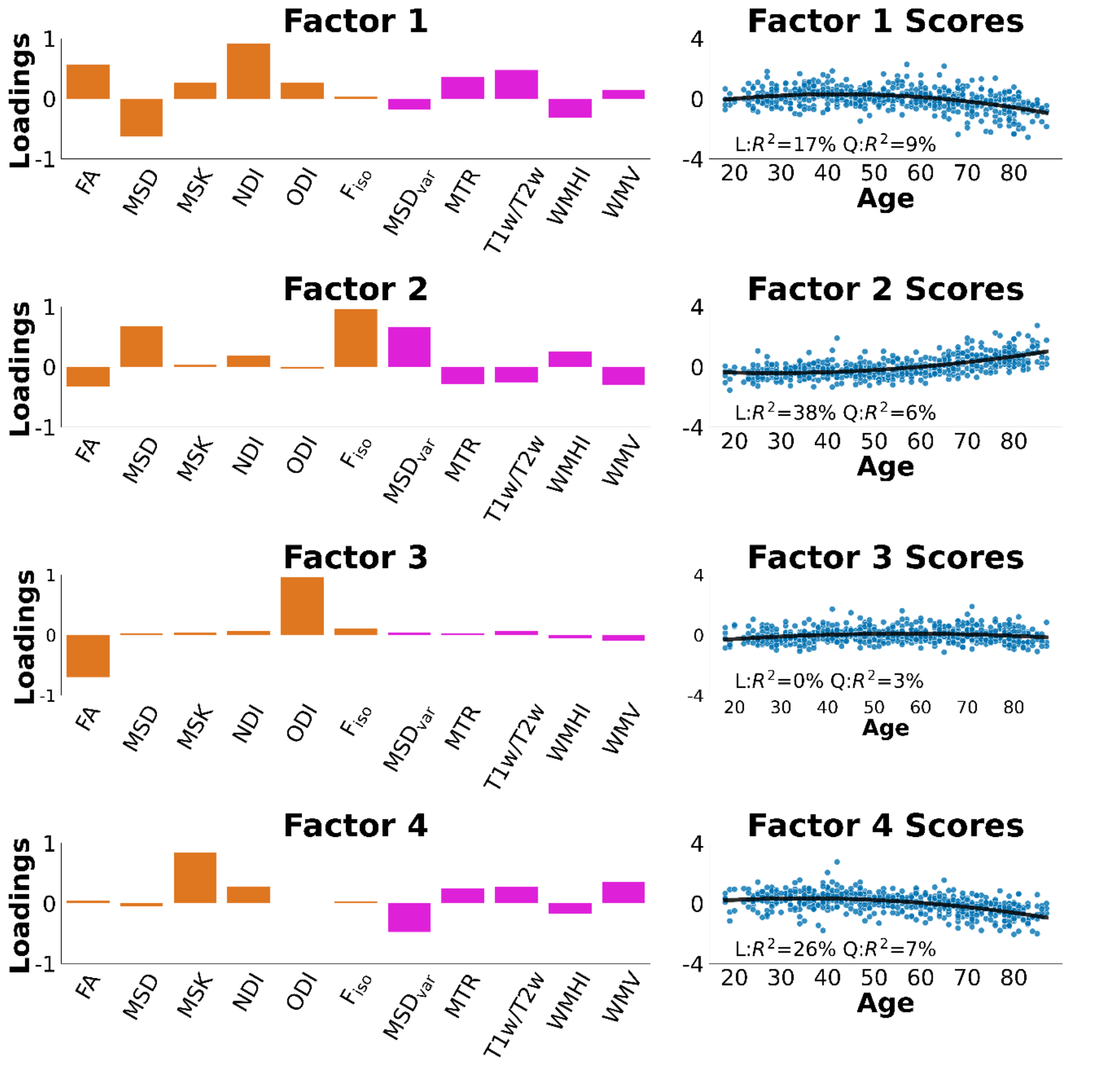

Supplementary Figure 1. *Factor loadings and scores for per ROI Factor analysis*. Here we show factor loadings and factor scores, when we performed factor analyses on data concatenated over ROIs rather than averaged over ROI as in main paper. Biggest difference in loadings was for Factor 3 which not also shows a negative loading on FA, which was not as strongly present in the main analyses.

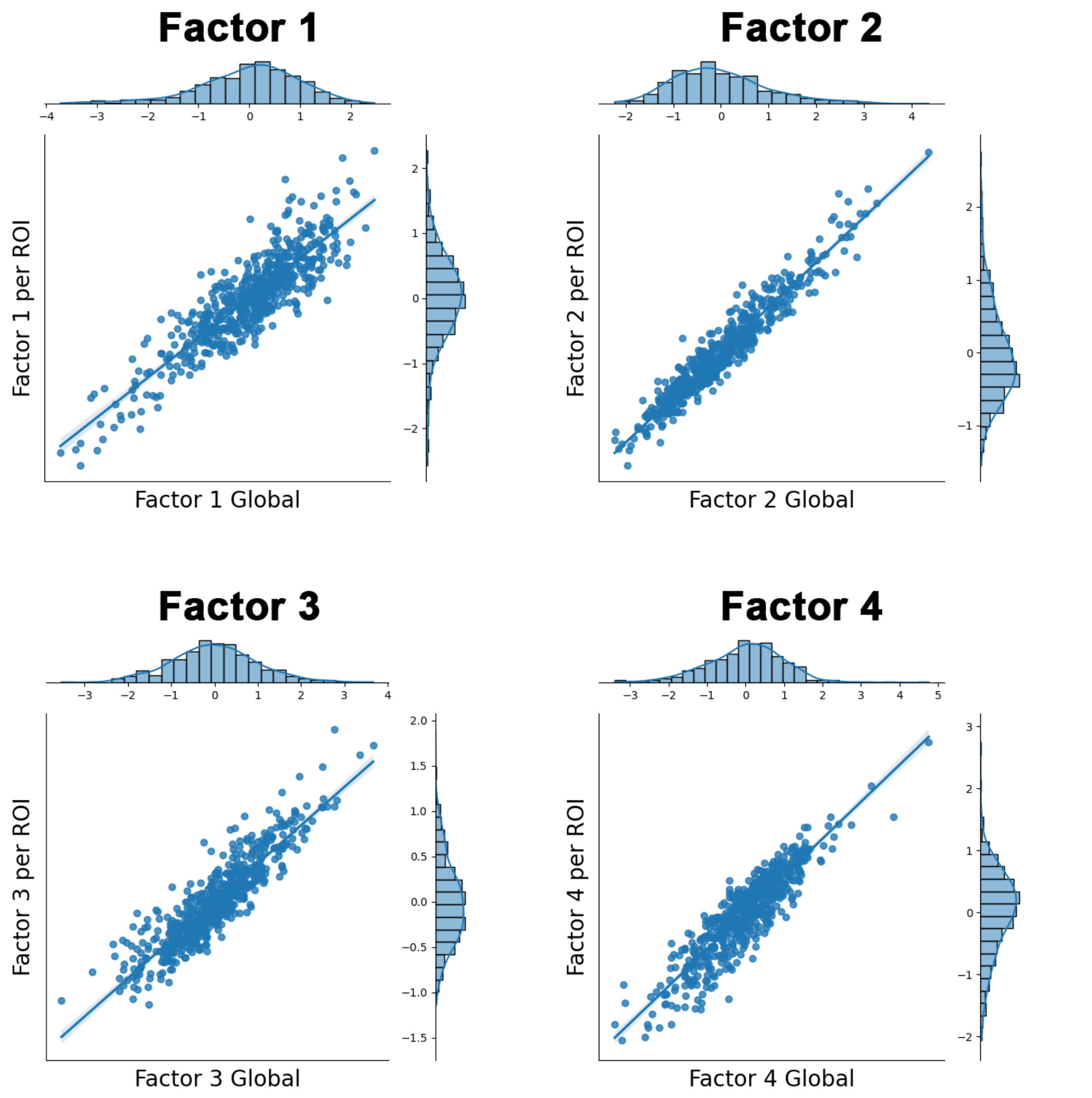

Supplementary Figure 2 Correlation between factor scores computed at whole brain level or concatenated per ROI.

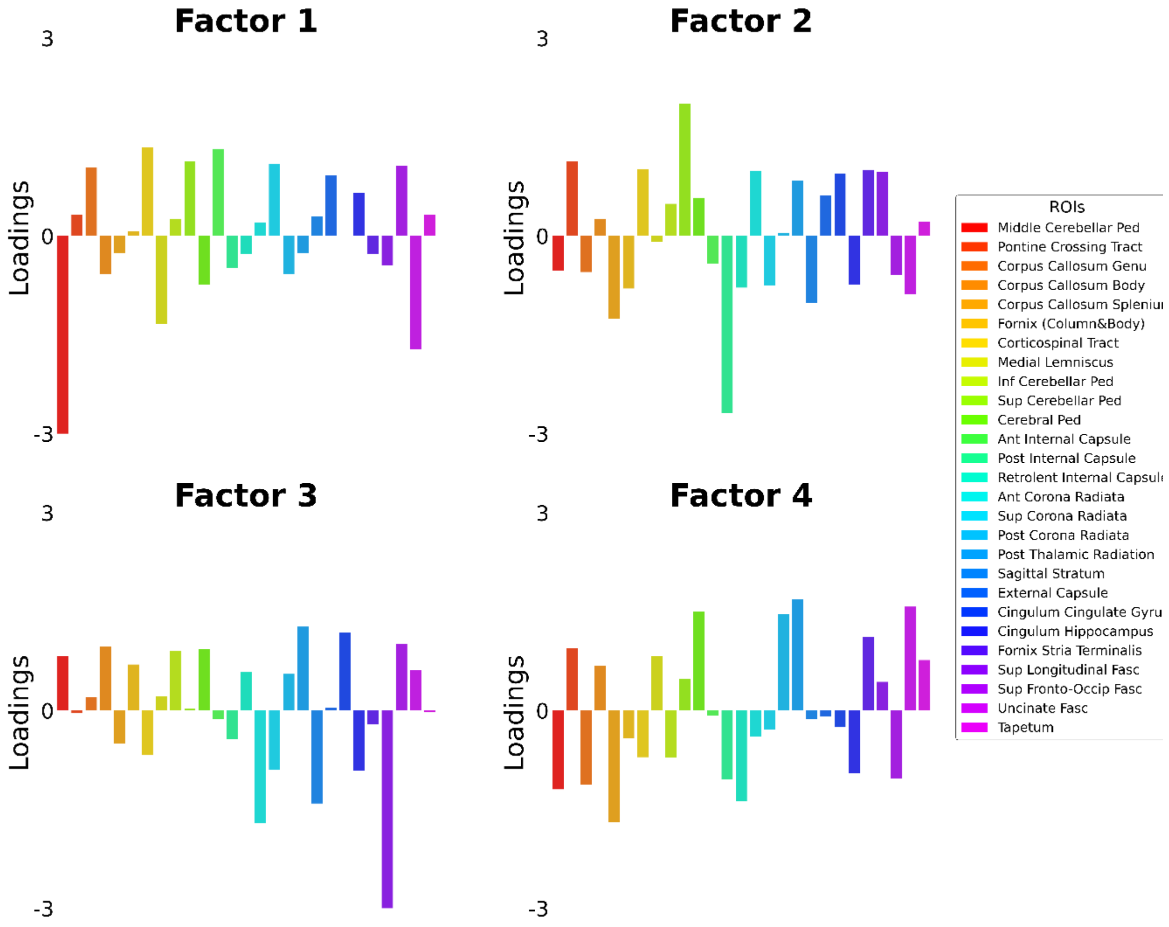

Supplementary Figure 3 *Loadings for the 27 ROIs used in the Factor analysis.* Here we show the average loading for each ROI. Loadings we z-scored for each factor.

Supplementary Table 2 *Association between each WM measure and age and sex.*

Measure	Age^1	Age^2	Sex	Age^1:Sex	Age^2:Sex
FA	-15.708 (p=0.000)	-3.776 (p=0.000)	-0.096 (p=0.004)	0.378 (p=0.677)	-0.051 (p=0.957)
MSD	19.513 (p=0.000)	9.049 (p=0.000)	0.009 (p=0.738)	-0.622 (p=0.374)	0.060 (p=0.934)
MSK	-13.006 (p=0.000)	-8.013 (p=0.000)	0.133 (p=0.000)	-0.271 (p=0.770)	1.536 (p=0.109)
NDI	-13.910 (p=0.000)	-8.101 (p=0.000)	-0.125 (p=0.000)	-0.053 (p=0.954)	0.616 (p=0.510)
ODI	-5.436 (p=0.000)	-9.179 (p=0.000)	-0.071 (p=0.069)	-0.644 (p=0.538)	0.293 (p=0.786)
F_{iso}	16.092 (p=0.000)	5.515 (p=0.000)	-0.123 (p=0.000)	-0.800 (p=0.361)	0.723 (p=0.423)
MSDvar	20.299 (p=0.000)	8.213 (p=0.000)	-0.087 (p=0.000)	-0.927 (p=0.166)	-0.096 (p=0.889)
MTR	-17.432 (p=0.000)	-5.652 (p=0.000)	0.025 (p=0.428)	-0.079 (p=0.925)	-0.922 (p=0.285)
T1/T2	-17.648 (p=0.000)	-9.618 (p=0.000)	-0.076 (p=0.008)	-0.237 (p=0.757)	0.580 (p=0.462)
WMHI	16.591 (p=0.000)	9.365 (p=0.000)	-0.015 (p=0.616)	0.450 (p=0.581)	0.001 (p=0.999)
WMV	-9.227 (p=0.000)	-10.186 (p=0.000)	0.016 (p=0.664)	2.092 (p=0.034)	1.374 (p=0.175)

Individual white measures and Cognition.

We examined how individual WM measures predicted cognition. To make sure the comparison is fair, we focused on the 570 participants that had data for all 11 WM measures. We predicted each individual cognitive measure from each individual WM measure while controlling for age and sex (DV ~ WM_measure + agepoly_1 + agepoly_2 + Sex). The results (Sup Fig 3) could guide future neurocognitive studies of ageing that need to choose a single measure of WM.

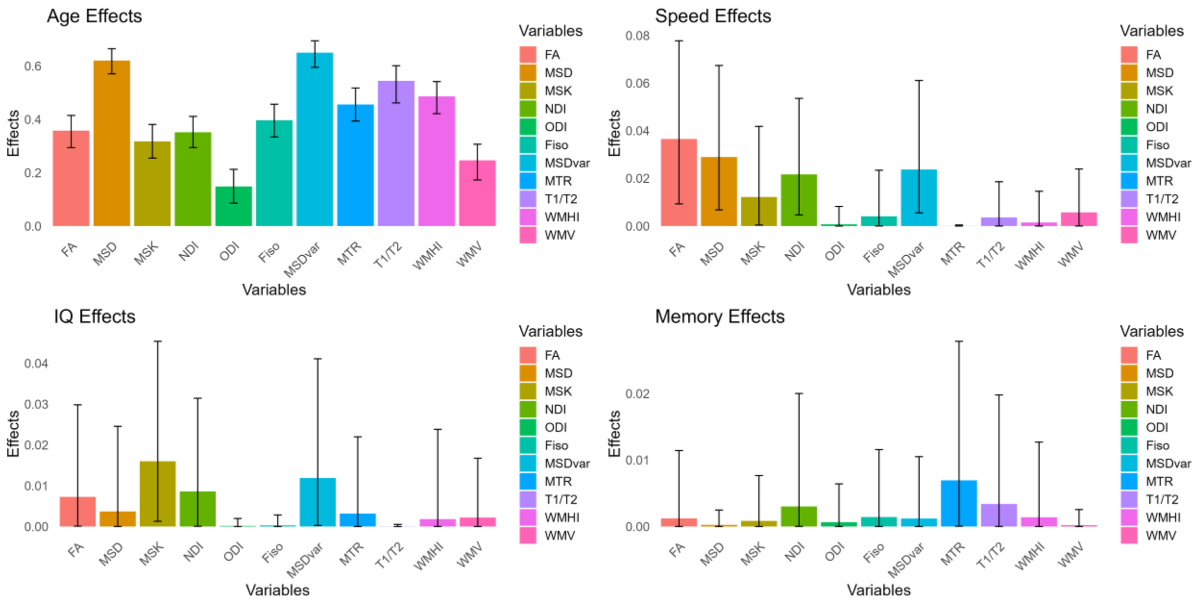

Supplementary Figure 4 *Effect sizes for each individual WM measure and age and cognition.* We show partial eta squared for each measure predicting each of the cognitive measures and cognition independently. We used 1000 bootstraps to show confidence interval over the effect size. As indicated by the factor analysis, kurtosis is strongest predictor of fluid intelligence, whereas FA, MSD and NDI are stronger predictors for processing speed.

Similar to Sup Fig 3 above, we show (Sup Fig 4) how well each of the latent cardiovascular factors predicted each individual WM measure (WM ~ LVF1 + LVF2 + LVF3 + agepoly_1 + agepoly_2 + Sex).

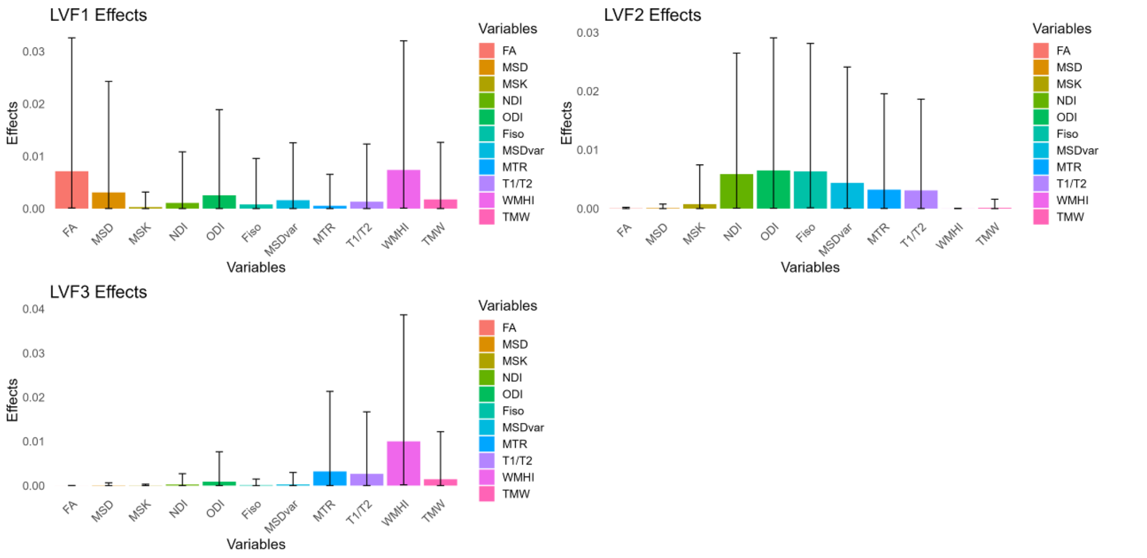

Supplementary Figure 5 Figure shows how strongly each latent cardiovascular factor is related to each individual white matter measure. We show partial eta square and bootstrapped confidence intervals.

Factor Analysis of cardiovascular measures

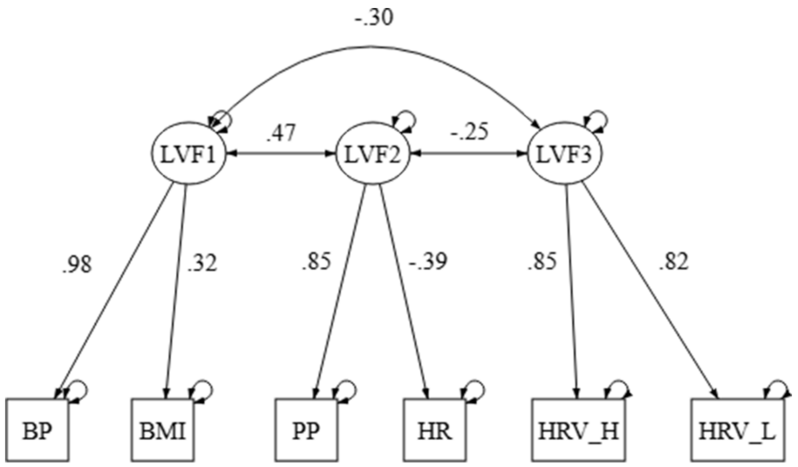

Supplementary Figure 6 *Latent Cardiovascular measures.* Figure shows main loadings for the three cardiovascular latent factors. Here we followed a previous report by King et al., (2023) and summarised our 6 cardio vascular measures into 3 related latent variables. LVF1 captured mainly static blood pressure (BP). LVF2 mainly loaded mainly on pulse pressure (PP). LVF3 loaded on both high and low frequency heart rate variability (HRV).

Correlation matrix for variables entering SEM models

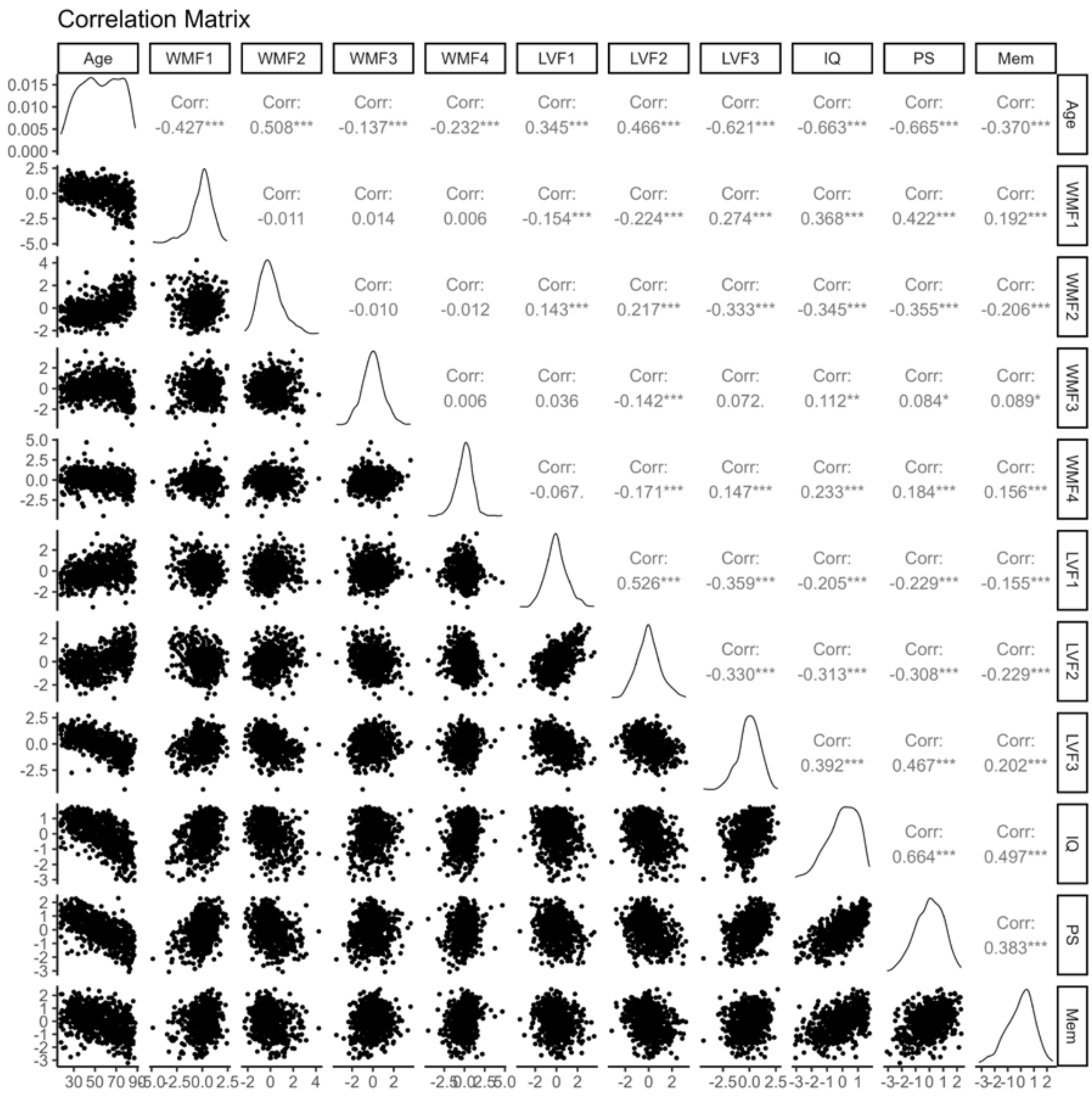

Supplementary Figure 7. Pairwise scatter plot and correlations for the main continuous variables that entered the SEM models. Each cell in the upper-triangle represents the pair-wise correlation between the relevant variables without adjusting for any confounds. *** $p < 0.001$

Cardiovascular and WM factors

For all path analyses we allowed the dependent variables to be correlated. This meant that covariance between IQ, PS and Memory was accounted for in the model, allowing us to examine unique paths between variables.

Supplementary Table 3 *Relationship between cardiovascular factors and white matter without accounting for Age.* Table shows estimated direct paths between vascular and white matter factor scores. We found that only pulse pressure factor (LVF2) was significantly related to the white matter factors representing free water content (WMF2).

lhs	op	rhs	est	se	*z*	*p*	ci.lower	ci.upper
WMF1	~	LVF1	0.00	0.04	0.11	.910	-0.08	0.09
WMF1	~	LVF2	-0.15	0.04	-3.44	.001***	-0.24	-0.07
WMF1	~	LVF3	0.22	0.04	5.51	< .001***	0.14	0.30
WMF2	~	LVF1	-0.03	0.04	-0.77	.440	-0.12	0.05
WMF2	~	LVF2	0.14	0.04	3.15	.002**	0.05	0.23
WMF2	~	LVF3	-0.30	0.04	-7.43	< .001***	-0.37	-0.22
WMF3	~	LVF1	0.16	0.05	3.52	< .001***	0.07	0.26
WMF3	~	LVF2	-0.21	0.05	-4.45	< .001***	-0.30	-0.12
WMF3	~	LVF3	0.06	0.04	1.51	.131	-0.02	0.15
WMF4	~	LVF1	0.06	0.05	1.21	.226	-0.04	0.15
WMF4	~	LVF2	-0.17	0.05	-3.53	< .001***	-0.26	-0.07
WMF4	~	LVF3	0.11	0.04	2.70	.007**	0.03	0.20

Cardiovascular and WM factors including age and sex

Supplementary Table 4 *Relationship between cardiovascular factors and white matter after accounting for Age and Sex.* Table shows estimated direct paths between vascular and white matter factor scores. We found that only pulse pressure factor (LVF2) was significantly related to the white matter factors representing free water content (WMF2).

lhs	op	rhs	est	se	*z*	*p*	ci.lower	ci.upper
WMF1	~	LVF1	-0.04	0.04	-0.98	.325	-0.12	0.04
WMF1	~	LVF2	-0.03	0.05	-0.61	.540	-0.12	0.06
WMF1	~	LVF3	0.01	0.04	0.17	.864	-0.08	0.09
WMF2	~	LVF1	0.01	0.04	0.22	.830	-0.07	0.09
WMF2	~	LVF2	-0.10	0.04	-2.31	.021*	-0.19	-0.02
WMF2	~	LVF3	-0.02	0.04	-0.55	.585	-0.11	0.06
WMF3	~	LVF1	0.09	0.05	1.85	.064	-0.01	0.18
WMF3	~	LVF2	-0.10	0.05	-1.96	.050*	-0.20	-0.00
WMF3	~	LVF3	-0.02	0.05	-0.44	.662	-0.12	0.07
WMF4	~	LVF1	0.02	0.05	0.51	.609	-0.07	0.11
WMF4	~	LVF2	-0.01	0.05	-0.25	.804	-0.11	0.09
WMF4	~	LVF3	0.00	0.05	0.03	.977	-0.09	0.10
WMF1	~	Age	-10.39	1.27	-8.18	< .001***	-12.88	-7.90
WMF1	~	QuadAge	-3.62	0.99	-3.65	< .001***	-5.56	-1.68
WMF1	~	Sex	-0.19	0.03	-5.35	< .001***	-0.25	-0.12
WMF2	~	Age	14.25	1.23	11.61	< .001***	11.84	16.65
WMF2	~	QuadAge	5.68	0.96	5.94	< .001***	3.81	7.55
WMF2	~	Sex	-0.14	0.03	-4.32	< .001***	-0.21	-0.08
WMF3	~	Age	-3.90	1.40	-2.79	.005**	-6.64	-1.16
WMF3	~	QuadAge	-7.21	1.09	-6.61	< .001***	-9.35	-5.07
WMF3	~	Sex	-0.09	0.04	-2.33	.020*	-0.16	-0.01
WMF4	~	Age	-6.15	1.41	-4.38	< .001***	-8.90	-3.40
WMF4	~	QuadAge	-4.85	1.10	-4.43	< .001***	-7.00	-2.71
WMF4	~	Sex	0.20	0.04	5.32	< .001***	0.13	0.28

WM and Cognitive factors not controlling for age

Supplementary Table 5 *Relationship between white matter factors and cognition without accounting for Age and Sex.* Table shows estimated direct paths between white matter factor scores and cognitive scores. We found that all paths between our white matter factors and cognitive measures were significant once we did not correct for age and sex effects.

lhs	op	rhs	est	se	*z*	*p*	ci.lower	ci.upper
IQ	~	WMF1	0.38	0.03	11.46	< .001***	0.32	0.45
IQ	~	WMF2	-0.35	0.03	-10.65	< .001***	-0.41	-0.28
IQ	~	WMF3	0.10	0.03	3.02	.003**	0.03	0.16
IQ	~	WMF4	0.24	0.03	7.30	< .001***	0.17	0.30
PS	~	WMF1	0.41	0.03	12.41	< .001***	0.34	0.47
PS	~	WMF2	-0.35	0.03	-10.57	< .001***	-0.41	-0.28
PS	~	WMF3	0.07	0.03	2.17	.030*	0.01	0.14
PS	~	WMF4	0.17	0.03	5.15	< .001***	0.11	0.24
Mem	~	WMF1	0.19	0.04	5.03	< .001***	0.11	0.26
Mem	~	WMF2	-0.20	0.04	-5.39	< .001***	-0.27	-0.13
Mem	~	WMF3	0.08	0.04	2.22	.026*	0.01	0.15
Mem	~	WMF4	0.15	0.04	4.05	< .001***	0.08	0.22

WM and Cognitive factors controlling for age and sex

Supplementary Table 6 *Relationship between white matter factors and cognition after accounting for Age and Sex.* Table shows estimated direct paths between white matter factor scores and cognitive scores. Fluid intelligence (IQ) was predicted by the first and fourth white matter factors capturing measures of white matter microstructure. Processing speed (PS) was predicted mainly by Factor 1, and showed a less robust association with Factor 2, representing free water content. Episodic Memory (Mem) was not significantly related to any of the white matter factors after accounting for age and sex effects.

lhs	op	rhs	est	se	*z*	*p*	ci.lower	ci.upper
IQ	~	WMF1	0.09	0.04	2.49	.013*	0.02	0.16
IQ	~	WMF2	-0.03	0.04	-0.88	.379	-0.11	0.04
IQ	~	WMF3	-0.02	0.03	-0.69	.488	-0.08	0.04
IQ	~	WMF4	0.10	0.03	3.02	.003**	0.03	0.16
PS	~	WMF1	0.17	0.04	4.74	< .001***	0.10	0.25
PS	~	WMF2	-0.08	0.04	-1.99	.047*	-0.16	-0.00
PS	~	WMF3	-0.01	0.03	-0.26	.799	-0.07	0.06
PS	~	WMF4	0.06	0.03	1.79	.074	-0.01	0.12
Mem	~	WMF1	0.04	0.04	1.00	.318	-0.04	0.13
Mem	~	WMF2	0.02	0.05	0.44	.663	-0.07	0.11
Mem	~	WMF3	0.02	0.04	0.64	.522	-0.05	0.10
Mem	~	WMF4	0.03	0.04	0.63	.528	-0.05	0.10
IQ	~	Age	-16.09	1.20	-13.40	< .001***	-18.44	-13.73
IQ	~	QuadAge	-3.74	0.94	-3.97	< .001***	-5.59	-1.89
IQ	~	Sex	-0.09	0.03	-2.88	.004**	-0.15	-0.03
PS	~	Age	-14.40	1.23	-11.67	< .001***	-16.82	-11.98
PS	~	QuadAge	-0.63	0.95	-0.67	.504	-2.49	1.22
PS	~	Sex	-0.05	0.03	-1.50	.134	-0.11	0.01
Mem	~	Age	-9.99	1.47	-6.80	< .001***	-12.87	-7.11
Mem	~	QuadAge	-2.03	1.15	-1.77	.077	-4.27	0.22
Mem	~	Sex	0.14	0.04	3.80	< .001***	0.07	0.21

WM and Cognitive factors controlling for age, sex and education

Supplementary Table 7 *Relationship between white matter factors and cognition after accounting for Age and Sex and Education.* Table shows estimated direct paths between white matter factor scores and cognitive scores. Fluid intelligence (IQ) was predicted by the first and fourth white matter factors capturing measures of white matter microstructure. Processing speed (PS) was predicted mainly by Factor 1, and showed a less robust association with Factor 2, representing free water content. Episodic Memory (Mem) was not significantly related to any of the white matter factors after accounting for age and sex effects.

lhs	op	rhs	est	se	*z*	*p*	ci.lower	ci.upper
IQ	~	WMF1	0.10	0.03	2.93	.003**	0.03	0.17
IQ	~	WMF2	-0.09	0.04	-2.37	.018*	-0.16	-0.01
IQ	~	WMF3	0.01	0.03	0.32	.751	-0.05	0.07
IQ	~	WMF4	0.09	0.03	2.91	.004**	0.03	0.15
PS	~	WMF1	0.18	0.04	4.97	< .001***	0.11	0.25
PS	~	WMF2	-0.11	0.04	-2.64	.008**	-0.18	-0.03
PS	~	WMF3	0.01	0.03	0.23	.820	-0.06	0.07
PS	~	WMF4	0.05	0.03	1.67	.095	-0.01	0.12
Mem	~	WMF1	0.06	0.04	1.28	.201	-0.03	0.14
Mem	~	WMF2	-0.02	0.05	-0.44	.663	-0.11	0.07
Mem	~	WMF3	0.05	0.04	1.31	.190	-0.02	0.12
Mem	~	WMF4	0.02	0.04	0.48	.628	-0.06	0.10
IQ	~	Age	-13.02	1.16	-11.23	< .001***	-15.30	-10.75
IQ	~	QuadAge	-2.14	0.89	-2.41	.016*	-3.89	-0.40
IQ	~	Sex	-0.09	0.03	-3.14	.002**	-0.14	-0.03
PS	~	Age	-12.89	1.26	-10.19	< .001***	-15.36	-10.41
PS	~	QuadAge	0.16	0.95	0.16	.870	-1.70	2.01
PS	~	Sex	-0.05	0.03	-1.56	.119	-0.11	0.01
Mem	~	Age	-7.60	1.49	-5.12	< .001***	-10.51	-4.69
Mem	~	QuadAge	-0.78	1.14	-0.69	.492	-3.00	1.45
Mem	~	Sex	0.14	0.04	3.87	< .001***	0.07	0.21
IQ	~	Education	0.30	0.03	9.89	< .001***	0.24	0.36
PS	~	Education	0.15	0.03	4.46	< .001***	0.08	0.22
Mem	~	Education	0.23	0.04	5.90	< .001***	0.16	0.31

Genetic effects on fluid intelligence

The PGS scores were not associated with any of the WM factors (see Sup. Fig. 2). IQ PGS was significantly related to Fluid Intelligence, as expected ($\beta = 0.17$, $t = 5.78$, $p < 0.001$, $\eta^2 = 0.06$). When we controlled for effect of PGS, most WMFs remained related to cognition (see Supp. Table 8). This implies that the WMFs are capturing brain-cognition associations not fully explained by chronological age or PGS scores for intelligence, such as environmental factors.

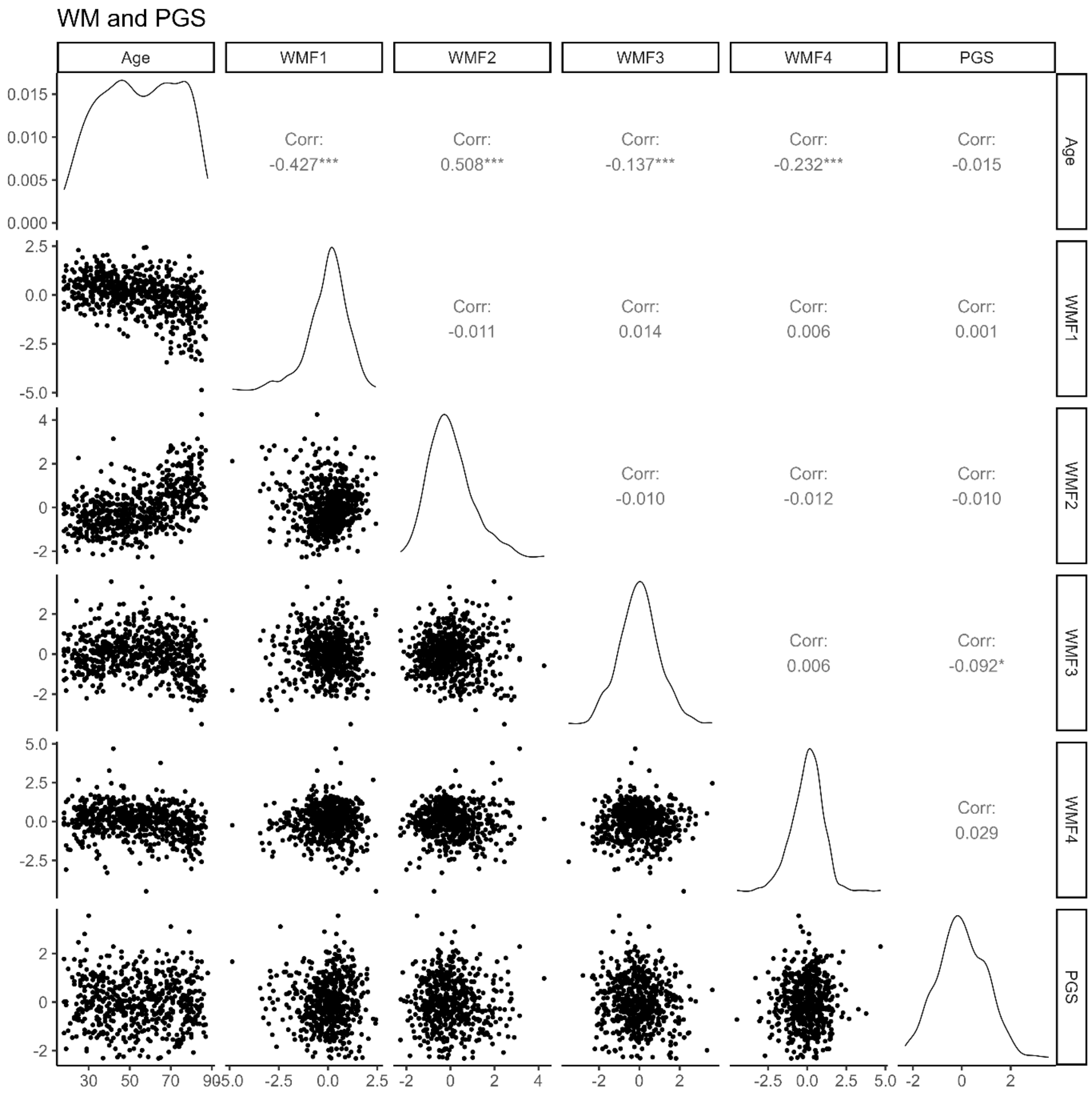

Supplementary Figure 8 *Correlation matrix between age, each of the 4 WM factors and PGS for cognitive ability.* Matrix shows PGS was not strongly related to any of the WM factors.

WM and Cognitive factors controlling for age and polygenic effects.

Supplementary Table 8 *Relationship between white matter factors and cognition controlling for polygenic effects (as well as Age and Sex).* Table shows estimated direct paths between WMFs and cognitive scores after accounting for age and polygenic effects. Fluid intelligence (IQ) was predicted by the first and fourth WMFs capturing measures of white matter microstructure. Processing speed (PS) was predicted mainly by Factor 1, but was not associated with Factor 2. Episodic Memory (Mem) was not significantly related to any of the WMFs after accounting for age and sex effects.

lhs	op	rhs	est	se	*z*	*p*	ci.lower	ci.upper
IQ	~	WMF1	0.09	0.04	2.53	.011*	0.02	0.17
IQ	~	WMF2	-0.02	0.04	-0.53	.597	-0.10	0.06
IQ	~	WMF3	0.00	0.03	0.00	.998	-0.07	0.07
IQ	~	WMF4	0.09	0.03	2.88	.004**	0.03	0.16
PS	~	WMF1	0.15	0.04	3.95	< .001***	0.08	0.23
PS	~	WMF2	-0.04	0.04	-0.95	.340	-0.12	0.04
PS	~	WMF3	0.02	0.03	0.61	.540	-0.05	0.09
PS	~	WMF4	0.05	0.03	1.48	.139	-0.02	0.12
Mem	~	WMF1	0.05	0.05	1.07	.286	-0.04	0.15
Mem	~	WMF2	0.01	0.05	0.17	.864	-0.09	0.11
Mem	~	WMF3	0.06	0.04	1.38	.167	-0.03	0.14
Mem	~	WMF4	0.02	0.04	0.52	.606	-0.06	0.11
IQ	~	Age	-15.90	1.27	-12.51	< .001***	-18.39	-13.41
IQ	~	QuadAge	-3.52	0.98	-3.60	< .001***	-5.44	-1.61
IQ	~	Sex	-0.08	0.03	-2.56	.010*	-0.14	-0.02
PS	~	Age	-15.56	1.31	-11.88	< .001***	-18.13	-13.00
PS	~	QuadAge	-0.44	1.01	-0.44	.660	-2.42	1.53
PS	~	Sex	-0.03	0.03	-0.86	.390	-0.09	0.04
Mem	~	Age	-8.66	1.66	-5.22	< .001***	-11.90	-5.41
Mem	~	QuadAge	-0.66	1.28	-0.52	.604	-3.16	1.84
Mem	~	Sex	0.15	0.04	3.70	< .001***	0.07	0.23
IQ	~	PGS	0.15	0.03	5.16	< .001***	0.10	0.21
PS	~	PGS	0.11	0.03	3.47	.001***	0.05	0.17
Mem	~	PGS	0.17	0.04	4.42	< .001***	0.10	0.25

Factor analysis for only diffusion metrics

Supplementary Table 9 *PCA for DWI metrics*. This table shows number of components identified for the same PCA analyses where we only used the 6 DWI measures. We show the variance explained (VE) by the last component (Last Comp) and the cumulative variance explained by the number of components retained.

Measures	N Measures	N Comp *ekf*	VE Last Comp	Cum VE
DWI	6	3	11.63	92.44

Multi-Group SEM models

Based on a reviewer suggestion, and in order to explore whether age might moderate some of the observed relationships we reported in the SEM models in the main paper, we ran a multi-group SEMs. We first split the sample into 3 approximately equally sized age groups (Group 1: N = 246; aged 18-45; Group 2: N = 235; aged 46-66; Group 3: N = 227; aged 67-88). We then ran models were we either equated the parameters across the 3 groups (e.g., the path coefficient between WMF1 to IQ is constrained to be invariant to age-group), referred to as a constrained model; or we allowed the association between WMF1 and IQ to differ across groups (e.g., the path coefficient between WMF1 to IQ could differ across age-groups), referred to as the unconstrained model. We then ran a model comparison between these two types of models, for both the SEM relating cardiovascular health to white matter and the SEM relating white matter to cognitive measures. The fit of the SEM relating LVF to WM was better for the constrained model (AIC = 6272; BIC=6565) compared to the unconstrained model (AIC=6280; BIC=6680), suggesting that the relationships were invariant to age. Similarly, we found better fit for the constrained (AIC=4188; BIC = 4402.3) vs unconstrained (AIC=4203; BIC=4524.5) model when we examined the association between WMF and Cognition, again supporting no age-moderation.
